# White Matter in Crisis: Oligodendrocytes and the Pathophysiology of Multiple Sclerosis

**DOI:** 10.3390/cells14181408

**Published:** 2025-09-09

**Authors:** Mario García-Domínguez

**Affiliations:** 1 Program of Immunology and Immunotherapy, CIMA-Universidad de Navarra, 31008 Pamplona, Spain; mgdom@unav.es; 2 Department of Immunology and Immunotherapy, Clínica Universidad de Navarra, 31008 Pamplona, Spain; 3 Centro de Investigación Biomédica en Red de Cáncer (CIBERONC), 28029 Madrid, Spain

**Keywords:** multiple sclerosis, demyelination, oligodendrocytes, differentiation, remyelination, inflammatory milieu, blood–brain barrier

## Abstract

Multiple sclerosis is a chronic, immune-mediated neurodegenerative disorder of the central nervous system, characterized by widespread demyelination, axonal injury, and progressive neurological impairment. The pathophysiology of multiple sclerosis involves complex interactions between immune cells and central nervous system resident cells, with oligodendrocytes (the myelin-producing glial cells) occupying a central role in both the disease’s onset and progression. Oligodendrocyte dysfunction, including diminished regenerative capacity, heightened vulnerability to inflammatory cytokines, and increased susceptibility to oxidative stress, contributes significantly to the failure of remyelination observed in chronic multiple sclerosis lesions. Key factors such as microglial activation, T-cell-mediated cytotoxicity, and altered signaling pathways affecting oligodendrocyte progenitor cell maturation are explored in depth. Some therapeutic strategies under investigation encompass the use of pharmacological agents, cell-based interventions, and modulation of both the extracellular matrix and the immune microenvironment. Advancing our understanding of oligodendrocyte biology, along with the intrinsic and extrinsic factors that impede effective remyelination, is critical for the development of innovative, targeted therapies aimed at attenuating neurodegeneration and enhancing long-term clinical outcomes in patients with multiple sclerosis.

## 1. Introduction

Multiple sclerosis (MS) is a chronic, autoimmune, and neurodegenerative pathology of the central nervous system (CNS) that constitutes one of the leading causes of non-traumatic neurological disability among young adults [1]. MS typically manifests between the ages of 20 and 40 and exhibits a marked female predominance, with a female-to-male ratio of approximately 3:1 [2,3]. This disease also shows the prevalence of significant geographical variability, being more commonly observed in populations residing at higher latitudes [4]. Despite decades of intensive research, the etiology of this disease remains understood, and its pathophysiology is shaped by an interplay between genetic susceptibility, environmental exposures (such as vitamin D deficiency, Epstein–Barr virus infection, and smoking), and dysregulated immune responses [5,6].

MS is characterized by a heterogeneous array of clinical manifestations. These clinical manifestations reflect the multifocal distribution of demyelination and axonal damage throughout the CNS, encompassing both cerebral and spinal regions [7]. Early symptoms include sensory disturbances (e.g., paresthesia and numbness), optic neuritis (resulting in visual loss and eye pain), and motor deficits like limb weakness or spasticity [8]. As the disease progresses, patients may develop ataxia, dysarthria, diplopia, and impaired coordination due to cerebellar and brainstem involvement [9]. Cognitive dysfunction, fatigue, and mood disorders, mainly depression and anxiety, are also regularly observed and may significantly impact quality of life [10]. Moreover, autonomic dysfunction can lead to bladder, bowel, and sexual disturbances [11].

This disease exhibits considerable heterogeneity in its clinical course, leading to the recognition of distinct subtypes. The most common form, relapsing–remitting MS (RRMS), is characterized by well-defined episodes of neurological dysfunction, followed by intervals of partial or complete recovery [12]. Over time, many RRMS patients transition to secondary progressive MS (SPMS), characterized by a progressive and irreversible accumulation of disability [13]. Primary progressive MS (PPMS), by contrast, manifests as progressive neurological deterioration from the onset, in the absence of relapses [14], whereas progressive relapsing MS (PRMS) combines steady progression with superimposed acute exacerbations [15]. Understanding these subtypes is essential for prognosis, management, and therapeutic decision-making [16].

**Figure 1:**
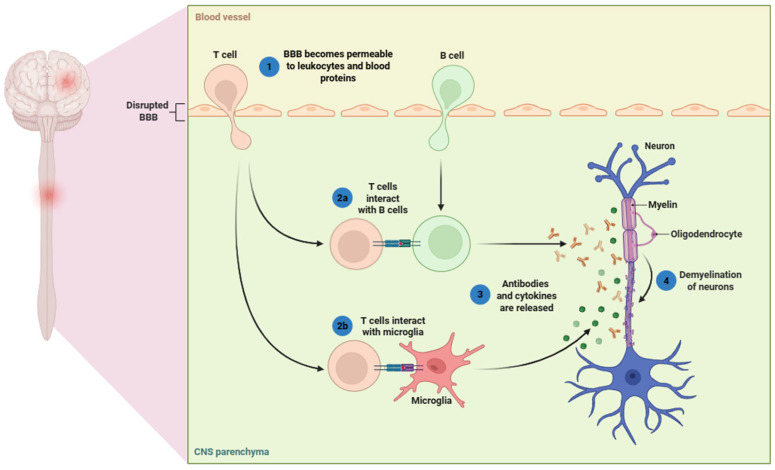
Disruption of the BBB permits the entry of several leukocytes (e.g., T cells and B cells), as well as other immune cell subsets such as monocytes, and neutrophils, together with blood-derived proteins, into the CNS parenchyma. This process involves the following steps: (1) T cells infiltrate the CNS through the permeabilized BBB; (2a) T cells interact with B cells, and (2b) with resident microglia, leading to immune activation; (3) activated immune cells release pro-inflammatory cytokines and antibodies; (4) these immune mediators contribute to oligodendrocyte injury and demyelination of neurons, a hallmark of MS. Abbreviations: BBB (blood–brain barrier) and CNS (central nervous system). Image adapted from BioRender.

Histopathologically, MS is characterized by focal demyelinated plaques in the white and gray matter of the brain and spinal cord, associated with inflammation, gliosis, axonal transection, and neurodegeneration [17,18]. These lesions are often accompanied by perivascular infiltration of white blood cells (mainly lymphocytes and monocytes), suggesting a breakdown of the blood–brain barrier (BBB) and entry of autoreactive immune cells into the CNS parenchyma (Figure 1) [19,20]. During the initial phase of MS, the clinical course is characterized by episodes of acute inflammation and demyelination, typically manifesting as relapses [21]. However, as the disease advances, neurodegenerative processes become the main contributors to disability progression, usually occurring independently of overt inflammatory activity [22].

Central to the disease process are oligodendrocytes, the myelinating glial cells of the CNS [23], which ensure rapid nerve conduction, provide strong trophic support to axons, and play a key role in metabolic coupling between neurons and glial cells [24,25]. In MS, oligodendrocytes are targeted by several immune-mediated mechanisms. Autoreactive CD4^+^ and CD8^+^ T cells, B cells, and activated microglia release pro-inflammatory cytokines (e.g., TNF-α, IFN-γ, and IL-1β), nitric oxide (NO), and reactive oxygen/nitrogen species (ROS/RNS, respectively) that compromise oligodendrocyte survival and function [26,27,28]. On the other hand, humoral mechanisms involving oligodendrocyte-specific autoantibodies may also play a crucial role in some MS subtypes [29].

Following demyelination, oligodendrocyte progenitor cells (OPCs), which are abundantly distributed within the adult CNS, are activated and mobilized toward demyelinated lesions in an effort to initiate repair. These cells proliferate, migrate, and differentiate into mature oligodendrocytes to facilitate remyelination [30]. However, in MS, this regenerative process often fails, especially in chronic lesions, where OPCs may persist in a quiescent state or fail to integrate into the demyelinated niche [31]. Some intrinsic and extrinsic factors are known to contribute to this remyelination failure. Among intrinsic barriers are epigenetic dysregulation, senescence, and altered transcriptional programs within the OPCs [32,33,34]. Extrinsic inhibitory signals include persistent inflammation, altered extracellular matrix composition (e.g., chondroitin sulfate proteoglycans), and dysregulation of key signaling pathways including Wnt, Notch, and BMP all of which can prevent OPC maturation and myelin sheath formation [35,36,37]. Compounding this scenario is the observation that chronic demyelination not only results in functional deficits but also renders axons vulnerable to degeneration [38]. The myelin sheath not only ensures efficient signal conduction but also contributes to axonal metabolism by supplying oligodendrocyte-derived lactate and pyruvate [39]. Therefore, the dysfunction (or loss) of oligodendrocytes leads to axonal metabolic stress, Ca^2+^ overload, and mitochondrial dysfunction, setting the stage for irreversible axonal loss and progressive neurological decline [40]. Although myelin has been shown to play protective roles in the pathogenesis of MS, evidence from Schäffner et al. [41] revealed that inflammatory conditions compromise this protection, rendering axons more vulnerable. Further investigations will be required to advance this line of research.

Although current disease-modifying therapies (DMTs) have significantly enhanced the clinical management of MS (primarily by reducing relapse frequency and limiting the formation of new lesions through modulation of peripheral immune activity), they have limited efficacy in preventing long-term neurodegeneration or facilitating remyelination [42]. This has prompted increasing interest in the development of neuroregenerative therapies aimed at selectively enhancing endogenous CNS repair mechanisms. Among these, strategies aimed at promoting oligodendrocyte survival, enhancing OPC recruitment and differentiation, and modulating the lesion microenvironment to favor remyelination have gained significant traction [43,44]. Emerging approaches include small-molecule modulators (e.g., clemastine fumarate, and benztropine), mAbs (e.g., opicinumab), and cell-based therapies involving OPC transplantation or manipulation of endogenous glial stem cells [45,46,47].

In this framework, an in-depth elucidation of the processes that govern oligodendrocyte functionality, viability, and regenerative capacity is crucial. This review aims to provide an integrative overview of oligodendrocyte pathology in MS, elucidating the factors underlying remyelination failure and the influence of the immune microenvironment on the regulation of glial repair mechanisms. The review also examines current and emerging therapeutic strategies aimed at restoring oligodendrocyte populations and re-establishing functional myelin within the damaged CNS. Ultimately, harnessing the regenerative potential of oligodendrocytes may facilitate the development of disease-modifying interventions that not only attenuate inflammation but also preserve neural integrity and enhance long-term clinical outcomes in individuals with MS.

## 2. Pathophysiology of Multiple Sclerosis

MS is characterized by an interplay between immune-mediated demyelination, oligodendrocyte injury, axonal degeneration, and glial dysfunction. While traditionally classified as an autoimmune disorder, growing evidence suggests that MS is also a disease of failed regeneration, in which neurodegeneration occurs in the absence of inflammatory responses, frequently in progressive disease stages. The subsequent sections delineate the cellular and molecular mechanisms underpinning MS pathophysiology, emphasizing immune dysregulation, myelin degradation, glial cell dynamics, and remyelination impairment. While the role of oligodendrocytes in MS is mentioned, a more comprehensive analysis will be provided in Section 3.

### 2.1. Peripheral Immune Activation and CNS Infiltration

The pathogenesis of MS arises from dysregulated activation of autoreactive immune cells in the periphery and their subsequent infiltration into the CNS. This process encompasses autoreactive T cell activation, B cell involvement, and mechanisms leading to BBB disruption, which collectively permit CNS immune entry and neuroinflammation. Moreover, molecular mimicry between Epstein–Barr virus (EBV) epitopes and myelin antigens promotes cross-reactive T cell activation, while EBV infection promotes epitope spreading and bystander activation, synergistically amplifying autoreactive clones and perpetuating the pathogenic immune response.

In summary, this section addresses the molecular mechanisms driving autoreactive T cell activation, the involvement of B cells, and the pathways responsible for BBB disruption, which together promote CNS immune cell infiltration and neuroinflammation.

#### 2.1.1. Activation of Autoreactive T Cells

Autoreactive CD4^+^ T lymphocytes, mainly Th1 and Th17 subsets, represent the cornerstone of MS immunopathogenesis. These cells recognize myelin-derived peptides presented in the context of MHC-II molecules on APCs, mainly dendritic cells (DCs), in peripheral lymphoid organs [48,49]. DCs process myelin antigens such as myelin basic protein (MBP), proteolipid protein (PLP), and myelin oligodendrocyte glycoprotein (MOG) [50], thereby presenting peptide-MHC-II complexes to naïve CD4^+^ T cells through their T cell receptor (TCR) [51]. Antigen recognition combined with CD80/CD86-CD28 costimulation is necessary for full T cell activation [52]. Concurrent engagement of PRRs like TLRs on DCs, triggered by PAMPs or DAMPs, induces the production and secretion of several pro-inflammatory cytokines (e.g., IL-12, IL-23, and IL-6), which skew naïve T cell differentiation toward the Th1 and Th17 lineages [53].

Th1 cells predominantly secrete IFN-γ, a cytokine that promotes macrophage activation and enhances the expression of MHC molecules and adhesion proteins on endothelial and glial cells, allowing immune cell infiltration and antigen presentation within the CNS [54,55]. Th17 cells secrete IL-17 (including IL-17A and IL-17F), IL-21, and IL-22, and GM-CSF, which are essential mediators of CNS inflammation [56]. IL-17A signals through the IL-17 receptor complex, leading to the activation of NF-κB and MAPK pathways, which induce chemokines such as CXCL1, CXCL2, and CCL20, recruiting neutrophils and exacerbating tissue injury [57]. IL-17 also disrupts tight junction integrity in brain endothelial cells by downregulating occludin and claudin-5 expression, thereby contributing to BBB permeability [58].

On the other hand, the generation of autoreactive T cells may be precipitated by molecular mimicry, whereby viral epitopes (particularly from EBV) share sequence or structural homology with myelin antigens, leading to cross-reactive T cell activation [59]. EBV infection has been shown to induce epitope spreading and bystander activation, further amplifying autoreactive clones [60].

#### 2.1.2. Role of B Cells and Autoantibodies

B lymphocytes contribute to MS pathogenesis through interrelated mechanisms beyond classical antibody production. B cells act as APCs by internalizing myelin antigens via their BCR, processing them, and presenting peptide fragments on MHC-II molecules to autoreactive CD4^+^ T cells, thereby facilitating T cell reactivation within the CNS [61,62,63].

**Figure 2:**
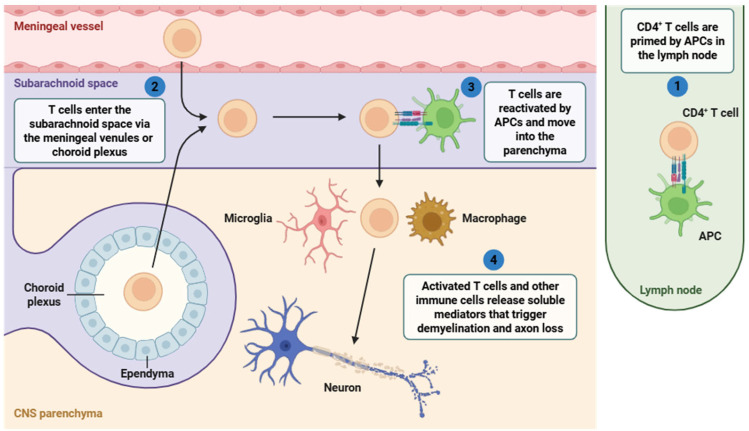
Mechanisms of T cell infiltration and CNS neuroinflammation in MS, illustrating how immune cells infiltrate the CNS primarily through the meninges and subarachnoid space, triggering localized inflammatory responses that contribute to disease progression. The following steps are involved: (1) CD4^+^ T cells are first primed by APCs in peripheral lymph nodes; (2) these activated CD4^+^ T cells then enter the CNS through the subarachnoid space (via meningeal vessels or the choroid plexus); (3) within the CNS, T cells are reactivated by local APCs and infiltrate the parenchyma; (4) subsequently, reactivated T cells (and others) such as microglia and macrophages release pro-inflammatory mediators that promote demyelination (4), contributing to neurodegeneration in MS. Abbreviations: CD4 (cluster of differentiation 4), APC (antigen-presenting cell), and CNS (central nervous system). Image adapted from BioRender.

In MS, ectopic lymphoid follicles resembling germinal centers are formed in the meninges. These structures support B cell clonal expansion, somatic hypermutation, and affinity maturation, enabling sustained intrathecal antibody synthesis [64]. Intrathecal IgGs, manifested as oligoclonal bands in CSF, targets CNS antigens, potentially including myelin and neuronal proteins [65]. Antibodies produced by B cells can induce demyelination via complement-dependent cytotoxicity (ADCC) [66]. The classical complement pathway is triggered by the binding of IgG to myelin components, which activates C1q, leading to the opsonization of targets by C3b and the subsequent formation of the membrane attack complex (MAC), which disrupts oligodendrocyte membranes and myelin sheaths (Figure 2) [67].

B cells also secrete several pro-inflammatory cytokines, such as LT-α and IL-6, which promote T follicular helper (Tfh) cell differentiation and enhance T cell effector functions [68]. IL-6, in particular, contributes to the survival of T cells and promotes Th17 polarization [69]. Additionally, Bregs, which usually exert immunosuppressive functions through IL-10 and TGF-β production, are functionally impaired in MS, thus reducing immune regulation and facilitating autoreactive inflammation [70].

#### 2.1.3. Disruption of the Blood–Brain Barrier

The BBB is a highly selective barrier that regulates the ingress of circulating cells and molecules into the CNS parenchyma. It is composed of endothelial cells, pericytes, and astrocytes, often including both inner and outer basement membranes, which collectively maintain CNS homeostasis and protect neural tissue from potentially harmful substances [71]. In MS, the BBB integrity is compromised, enabling leukocyte infiltration and initiation of CNS inflammation [20]. Molecularly, this breakdown is mediated by upregulation of several adhesion molecules on brain microvascular endothelial cells, including VCAM-1, ICAM-1, and selectins (such as P- and E-selectin) [72,73]. These molecules facilitate the multistep leukocyte adhesion cascade by interacting with integrins like VLA-4 and LFA-1 [74,75].

Simultaneously, several pro-inflammatory cytokines (TNF-α, IFN-γ, and IL-1β) stimulate endothelial cells to produce MMPs, particularly MMP-2 and MMP-9 [71]. These proteolytic enzymes degrade extracellular matrix components and tight junction proteins including claudin-5, occludin, and the zonula occludens family (e.g., ZO-1), disrupting the endothelial barrier [76]. The consequent loss of tight junction integrity increases paracellular permeability, allowing the transmigration of autoreactive T cells, B cells, monocytes, and neutrophils into perivascular and parenchymal CNS compartments [77]. Several chemokines secreted by activated microglia, astroglia, and endothelial cells (e.g., CCL2, CCL5, CXCL10, and CXCL12) create a chemotactic gradient that guides leukocyte extravasation and migration [78]. Leukocyte diapedesis involves signaling through endothelial receptors such as PECAM-1 and JAMs, facilitating transmigration across the endothelial basal lamina [79,80].

Finally, oxidative stress induced by infiltrating immune cells and activated by CNS glia generates ROS and RNS, thereby exacerbating endothelial injury and impairing BBB integrity [81].

### 2.2. Inflammatory Cascade Within the CNS

The inflammatory response within the CNS following the breach of the BBB represents a complex interplay between infiltrating peripheral immune cells and resident glial populations. This neuroinflammatory milieu is orchestrated through a cascade of molecular signaling events that perpetuate tissue damage and disrupt homeostatic neuronal-glial interactions. Upon infiltration of peripheral immune cells into the CNS, several types of glial cells (microglia and astroglia) are activated, functioning as the principal innate immune effectors within the parenchyma [82]. This activation initiates a pro-inflammatory cascade that amplifies neuroimmune signaling and contributes to tissue damage and demyelination.

Resting microglia, exhibiting a ramified morphology under homeostatic conditions, undergo morphological and functional transformation into an amoeboid (activated) state in response to PAMPs and/or DAMPs [83]. This activation is predominantly mediated by PRRs, such as TLRs (mainly TLR4) and NLRs [84,85]. Engagement of TLRs leads to downstream signaling via MyD88-dependent and TRIF-dependent pathways, culminating in the activation of transcription factors including NF-κB and IRF3 [86]. The transcriptional activation results in the secretion of pro-inflammatory mediators such as IL-1β, IL-6, and TNF-α [87]. Microglia also generate ROS through NADPH oxidase (NOX2) activation and release NO through the upregulation of inducible iNOS, both of which contribute to oxidative stress and neuronal injury [88,89].

Concurrently, astrocytes respond to inflammatory stimuli and undergo reactive astrogliosis, characterized by hypertrophy, upregulation of GFAP, and increased secretion of several bioactive molecules [90]. Activated astrocytes release S100β, a Ca^2+^-binding protein that operates as a DAMP, and amplifies microglial activation through RAGE signaling [91]. Moreover, astrocytes secrete IL-33, which has dual roles in tissue repair and immune activation depending on the context and receptor engagement [92]. Astrocytes also synthesize and deposit extracellular matrix components, particularly CSPGs, which are crucial components of the glial scar [93]. CSPGs, including neurocan, brevican, and versican, inhibit axonal regeneration and remyelination by interacting with neuronal receptors such as PTPσ and LAR, thereby creating a non-permissive environment for recovery [94,95].

Finally, several pro-inflammatory cytokines within the CNS microenvironment exert direct cytotoxic and regulatory effects on oligodendrocytes and their OPCs, thereby contributing to demyelination and impairing the endogenous capacity for remyelination [96]. These effects are mediated through both apoptotic signaling pathways and the inhibition of OPC maturation, ultimately disrupting myelin sheath [97].

## 3. The Role of Oligodendrocytes in Multiple Sclerosis

### 3.1. Oligodendrocyte Apoptosis and Necroptosis

In MS, the loss of oligodendrocytes is a critical pathological event that precedes and accompanies demyelination, axonal degeneration, and ultimately, neurological disability [98]. Demyelination in MS results from immune, oxidative, and apoptotic mechanisms, in which CD8^+^ T cells target MHC-I-presenting oligodendrocytes and generate caspase-dependent apoptosis through perforin and granzyme B cytotoxicity [99,100]. Additionally, oligodendrocyte degeneration not only leads to myelin loss but also results in the release of DAMPs, which amplify immune activation and perpetuate chronic inflammation within the CNS [101]. Two principal modes of programmed cell death, known as apoptosis and necroptosis, have been associated with the demise of oligodendrocytes within MS lesions, each governed by distinct molecular signaling cascades and contributing to lesion pathology depending on the disease stage and inflammatory milieu.

**Figure 3:**
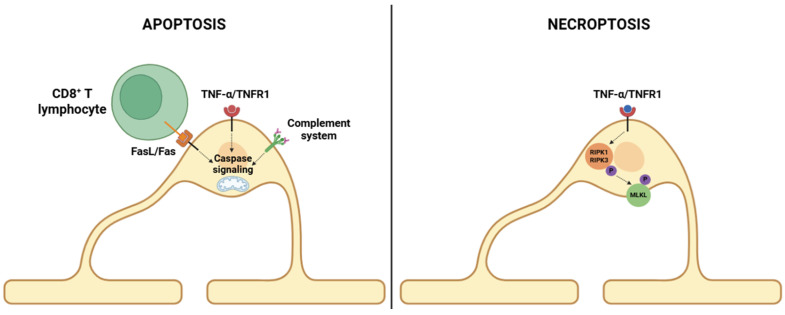
Mechanisms of apoptosis and necroptosis in oligodendrocytes. Left (apoptosis pathway): CD8^+^ T lymphocytes induce apoptosis in oligodendrocytes through FasL/Fas interactions. Furthermore, oligodendrocytes express TNFR1, enabling them to detect TNF-α present in the inflammatory milieu. The complement system can trigger apoptosis. Right (necroptosis pathway): When caspase activity is inhibited, TNF-α/TNFR1 engagement activates RIPK1 and RIPK3, leading to MLKL phosphorylation. Activated MLKL disrupts plasma membrane integrity, resulting in cell death. Abbreviations: CD8 (cluster of differentiation 8), FasL (Fas ligand), Fas (Fas receptor), TNF-α (tumor necrosis factor alpha), TNFR1 (tumor necrosis factor receptor 1), RIPK1 (receptor-interacting protein kinase 1), RIPK3 (receptor-interacting protein kinase 3), and MLKL (mixed lineage kinase domain-like protein). Image created with BioRender.

Apoptosis (Figure 3), a form of regulated cell death characterized by cellular shrinkage, chromatin condensation, and caspase activation, plays a prominent role in early MS lesions, particularly in so-called pattern III lesions, which are thought to reflect primary oligodendrogliopathy [102]. Oligodendrocytes enduring apoptosis show classic features like DNA fragmentation, mitochondrial outer membrane permeabilization (MOMP), and activation of executioner caspases, mainly caspase-3 and caspase-9 [103,104]. These events are often initiated by extrinsic death receptor signaling, via the Fas/FasL axis and TNFR1, both of which are upregulated in active MS lesions [105]. Engagement of these receptors recruits adaptor proteins such as FADD and procaspase-8 to form the death-inducing signaling complex (DISC), leading to the downstream activation of the caspase cascade [106]. In addition, intrinsic apoptotic pathways are strongly activated in response to intracellular stressors, such as glutamate excitotoxicity, oxidative damage, and endoplasmic reticulum (ER) stress [98]. A central pathway driving apoptotic processes involves the formation of the membrane attack complex (MAC), which is initiated by the binding of autoantibodies to MBP and/or MOG proteins [107,108,109]. This event activates the complement system and compromises oligodendrocyte membrane integrity, leading to an influx of Ca^2+^ ions that triggers the activation of apoptotic caspases [67]. These alterations are further accompanied by increased Na^+^ influx and K^+^ efflux, which compromise membrane potential and induce osmotic imbalance, ultimately promoting oligodendrocyte death [110,111]. Moreover, these autoantibodies mediate ADCC through engagement of Fcγ receptors on NK cells and macrophages [76].

In contrast, necroptosis (Figure 3) is increasingly recognized as a vital contributor to oligodendrocyte death, particularly in inflammatory conditions where caspase activity is inhibited or overwhelmed [112]. Necroptosis is orchestrated by receptor-interacting protein kinases RIPK1 and RIPK3, and the mixed lineage kinase domain-like protein (MLKL) [113,114]. In the context of MS, involvement of TNFR1 in the absence (and/or inhibition) of caspase-8 activity allows the phosphorylation and activation of the receptor-interacting protein kinases RIPK1 and RIPK3 [112]. Ultimately, this complex phosphorylates MLKL promotes its oligomerization and subsequent translocation to the plasma membrane, where it disrupts membrane integrity and leads to lytic cell death [115]. Oligodendrocytes within active MS lesions have been shown to express phosphorylated MLKL, supporting the involvement of necroptosis in disease pathology [116].

Despite this progressive decline, the surviving mature oligodendrocytes demonstrate notable resilience and attempt to protect neural function through the active synthesis and repair of myelin sheaths [117]. These surviving cells engage in a variety of compensatory mechanisms, including upregulation of myelin-associated proteins, metabolic adaptation to stress conditions, and close interaction with neighboring neurons and OPCs to promote localized remyelination [118]. Nevertheless, the effectiveness of this endogenous remyelination process is frequently constrained by the adverse conditions of the lesion microenvironment [119].

### 3.2. Impaired Oligodendrocyte Precursor Cell Differentiation

In MS, the failure of remyelination is a pathological feature of chronic demyelinated lesions and is strongly associated with the impaired differentiation of OPCs into mature oligodendrocytes [120]. Although OPCs are efficiently recruited to sites of demyelination, driven by chemotactic and mitogenic factors such as PDGF-A, FGF-2, and CXCL12, their ability to complete the full differentiation program is frequently compromised, leading to persistent demyelination, axonal vulnerability, and neurodegeneration [35]. This failure is not due to a strong depletion of the OPC pool but rather, reflects a molecular blockade that prevents progression through the critical stages of lineage commitment, myelin gene expression, and morphological maturation [31].

At the molecular level, both cell-intrinsic and extrinsic factors contribute to this differentiation arrest. Epigenetically, demyelinated lesions in MS exhibit a repressive chromatin landscape that inhibits the transcription of essential myelin-related genes [121]. Key transcription factors such as MYRF, SOX10, OLIG1, and OLIG2 are subject to transcriptional silencing through histone modifications including H3K27me3, a mark placed by the PRC2 via its catalytic subunit EZH2 [122,123]. Concurrently, there is a strong dysfunction of HDACs, particularly HDAC1 and HDAC2, which are normally required to remove acetyl groups and facilitate chromatin condensation necessary for oligodendrocyte lineage progression [124]. Moreover, alterations in DNA methylation patterns have been shown in OPCs within MS lesions. Notably, the loss of activity of some DNA methyltransferases (DNMTs) impairs remyelination efficiency in experimental MS models [125]. On the other hand, some post-transcriptional mechanisms contribute to the differentiation blockade in MS, as evidenced by the downregulation of miR-219 and miR-338, two microRNAs that facilitate oligodendrocyte maturation by suppressing transcriptional inhibitors like Hes5 and Sox6, thereby disrupting the gene expression control required for lineage progression [126,127].

Several developmental signaling pathways are aberrantly reactivated in the demyelinated CNS environment and act as potent inhibitors of OPC differentiation. One of the most prominent is the canonical Wnt/β-catenin pathway [128]. In MS, aberrant upregulation of Wnt ligands results in the stabilization and nuclear translocation of cytoplasmic β-catenin, which interacts with the TCF7L2 transcription factor [129]. This complex suppresses the transcription of key pro-differentiation genes such as MYRF and MBP, while concurrently promoting the expression of ID2 and ID4, which act as dominant negative inhibitors of basic helix-loop-helix (bHLH) transcription factors essential for oligodendrocyte maturation [130]. Conversely, activation of Notch1 on OPCs results in proteolytic cleavage and subsequent nuclear translocation of the Notch intracellular domain (NICD), which interacts with RBP-Jκ to drive the expression of transcriptional repressors such as Hes1 and Hes5, which suppress some pro-differentiation genes [131]. Further inhibition arises from BMPs, particularly BMP4, which is upregulated in the MS lesion milieu [132].

On the other hand, the inflammatory environment in MS lesions initiates additional layers of complexity that exacerbate the block in OPC differentiation [133]. IFN-γ activates STAT1 signaling in OPCs, inducing the expression of class II transactivator (CIITA) and MHC-II, functionally reprogramming OPCs into immunogenic, non-myelinating phenotypes [134]. TNF-α, notably in its soluble form (sTNF), signals through TNFR1 to activate NF-κB pathways, leading to pro-apoptotic and anti-differentiation effects in OPCs [135]. Simultaneously, IL-1β and IL-6 activate NF-κB and STAT3 pathways, respectively, both of which converge on downregulation of transcriptional regulators like SOX10 and NKX2.2, further suppressing the differentiation program in OPCs [136,137].

The extracellular matrix within demyelinated lesions undergoes extensive remodeling that presents both physical and biochemical barriers to OPC maturation [36]. CSPGs, which accumulate in MS lesions, engage receptors such as PTPσ and LAR on OPCs and activate intracellular RhoA/ROCK signaling, which impairs cytoskeletal reorganization necessary for process extension and myelin membrane formation [138,139]. Similarly, fibronectin aggregates (aFn), which persist in chronic lesions due to impaired matrix turnover, interfere with integrin signaling and cytoskeletal dynamics, and sequester pro-differentiation growth factors, thus depriving OPCs of essential inputs [140]. Other extracellular matrix molecules like hyaluronan and tenascin-C also contribute to this inhibitory environment [141,142]. Hyaluronan interacts with CD44 on OPCs, leading to intracellular signaling cascades that inhibit maturation [141], while tenascin-C has been demonstrated to interfere with integrin-mediated adhesion and signaling pathways on OPCs [142].

### 3.3. Mitochondrial Dysfunction and Energy Failure

In MS, mitochondrial dysfunction and the resulting bioenergetic failure are increasingly recognized as central contributors to the pathogenesis of both the inflammatory and progressive phases of the disease [143]. In addition to immune-mediated injury, activated microglia and infiltrating macrophages release high levels of ROS and RNS. These reactive species induce oxidative stress, resulting in lipid peroxidation, protein nitration, and destabilization of myelin [88,89]. The cumulative effect of immune and oxidative damage compromises the integrity of compact myelin, rendering it more susceptible to phagocytic clearance [144].

Oligodendrocytes are vulnerable to mitochondrial insults due to the high energy requirements necessary for synthesizing, compacting, and maintaining the multilamellar myelin sheath [145]. Under physiological conditions, oligodendrocytes rely primarily on OXPHOS to meet their ATP demands [146]. This process is orchestrated through the mitochondrial electron transport chain (ETC), embedded in the inner mitochondrial membrane, where electrons are shuttled from NADH and FADH_2_ to molecular O_2_ through complexes I (NADH:ubiquinone oxidoreductase), II (succinate dehydrogenase), III (cytochrome bc_1_ complex), and IV (cytochrome c oxidase), driving H^+^ translocation and creating an electrochemical gradient. This proton motive force powers ATP synthase, enabling the phosphorylation of ADP to ATP [147].

In the context of MS, several studies have reported profound defects in ETC activity, particularly affecting complex I and complex IV, both of which are sensitive to oxidative and nitrosative stress. Reduced activity of these mitochondrial respiratory chain components compromises proton gradient formation, disrupts the mitochondrial membrane potential, and ultimately impairs ATP synthesis through OXPHOS [148,149]. This metabolic collapse is especially detrimental in oligodendrocytes, where ATP is fundamental not only for membrane biosynthesis and vesicular trafficking but also for the operation of ion pumps like Na^+^/K^+^-ATPase, which are essential for maintaining osmotic balance and ionic gradients during neuronal signaling [150].

Defective electron transfer within the ETC leads to electron leakage, particularly at complexes I and III, resulting in the overproduction of superoxide anion (O_2_^−^), a key ROS [151]. O_2_^−^ is converted by mitochondrial manganese superoxide dismutase (MnSOD) into hydrogen peroxide (H_2_O_2_), which can further react via Fenton chemistry in the presence of Fe^2+^ to generate hydroxyl radicals (•OH), among the most cytotoxic ROS [152]. These ROS oxidize mitochondrial lipids, like cardiolipin (a critical phospholipid of the inner mitochondrial membrane that anchors cytochrome c), disrupting mitochondrial membrane integrity on oligodendrocytes [153]. Furthermore, mtDNA, that directs the synthesis of 13 essential subunits of the ETC, is vulnerable to oxidative damage due to its close proximity to sites of ROS generation, absence of protective histone proteins, and reduced capacity for DNA repair [154,155]. 8-OHdG accumulate in mtDNA, leading to point mutations, deletions, and impaired transcription of ETC subunits, thereby perpetuating mitochondrial dysfunction in a feed-forward loop [156].

Simultaneously, neuronal ATP depletion impairs energy-dependent ion pump activity, intensifying ionic dysregulation and further destabilizing axonal integrity [157]. The compromised axonal cytoskeleton, including microtubules and neurofilaments, disrupts axonal transport mechanisms critical for the delivery of organelles and synaptic vesicles, culminating in distal axonal degeneration [158]. Moreover, demyelinated axons show increased vulnerability to glutamate excitotoxicity through upregulated AMPA/kainate receptors, which further exacerbates Ca^2+^-mediated injury [159].

Finally, in MS, activated microglia and infiltrating immune cells promote the upregulation of iNOS expression in several resident glial cells (e.g., astroglia) [160]. This results in excessive levels of NO, which reacts with superoxide to form peroxynitrite (ONOO^−^), a potent nitrating agent that modifies tyrosine residues on mitochondrial proteins, including components of complex I and complex IV, further impairing their activity [161].

### 3.4. Disruption in the Formation of Myelin Proteins

The lesion microenvironment in MS is strongly characterized by extracellular matrix components such as CSPGs, fibronectin aggregates, and hyaluronan, which interact with OPC receptors (e.g., PTPσ and CD44) to activate some intracellular signaling cascades that inhibit cytoskeletal dynamics and myelin protein synthesis [162,163]. The disruption of myelin protein expression is a pathological process that plays a crucial role in the development of MS [164]. This phenomenon results from a cascade of molecular events that collectively impair the regulatory networks guiding myelin protein synthesis, processing, and localization of several structural proteins of the myelin sheath, including MBP, PLP1, MAG, and MOG [165]. Under physiological conditions, the expression of myelin-associated proteins is tightly controlled during oligodendrocyte lineage progression and myelination, ensuring proper compaction of axons [166]. However, in MS, inflammatory and cell-intrinsic mechanisms interfere with their expression at multiple levels. In addition to the aforementioned mechanisms, there are additional pathways that further inhibit the transcription and translation of proteins involved in myelinogenesis.

In the context of translational regulation and proteostasis within MS lesions, converging pro-inflammatory cytokines and metabolic perturbations elicit strong inhibition of the integrated stress response (ISR) [167], accompanied by robust activation of ER stress pathways [168], both fundamental in maintaining cellular homeostasis under adverse conditions. Activation of the PERK arm of the UPR leads to the phosphorylation of eIF2α protein [98]. This phenomenon initiates a global attenuation of cap-dependent mRNA translation by inhibiting the guanine nucleotide exchange factor eIF2B, effectively reducing the initiation of protein synthesis [169]. This repression affects proteins with high biosynthetic demand and complex folding requirements, including MBP and PLP1, which are essential for myelin sheath maintenance and integrity [170].

Simultaneously, the accumulation of misfolded myelin proteins within the ER lumen functions as a potent stimulus for the activation of the other two UPRs, inositol-requiring enzyme 1 alpha (IRE1α) and activating transcription factor 6 (ATF6) [171,172]. IRE1α, upon activation, induces oligomerization and autophosphorylation, which in turn activates its endoribonuclease function to process XBP1 mRNA [173]. Spliced XBP1 mRNA functions as a transcriptional activator that upregulates several genes that encode ER chaperones, like BiP/GRP78 and GRP94, as well as some components of the ERAD pathway, thereby contributing to the restoration of ER protein-folding homeostasis [174]. At the same time, ATF6 translocates from the ER to the Golgi Apparatus, where it is proteolytically cleaved to release its cytosolic domain, which acts as a transcription factor enhancing the expression of some chaperones and ER quality control machinery [175]. However, prolonged or excessive UPR signaling results in sustained translational repression that suppresses synthesis of myelin proteins, further impairing remyelination [176].

Finally, at the post-translational level, aberrations in myelin protein processing critically undermine protein function. Aberrant N-glycosylation of MOG in the Golgi impairs its folding and disrupts its trafficking to the oligodendrocyte membrane [177].

## 4. Therapeutic Strategies Targeting Oligodendrocytes

### 4.1. Promoting OPC Differentiation

A fundamental approach to promoting remyelination in demyelinating disorders involves the activation and directed differentiation of OPCs. The transition of OPCs from a proliferative to a differentiated state is tightly regulated by several key molecular signaling pathways, each exerting specific temporal and context-dependent effects (Table 1).

One pivotal pathway is the Wnt/β-catenin signaling cascade, which plays a dual regulatory role (Table 1) [178]. Transient activation of canonical Wnt signaling (mediated via Wnt ligands binding to Frizzled receptors and co-receptor LRP5/6) leads to stabilization of β-catenin, its translocation into the cell nucleus, and subsequent transcriptional activation of several target genes (such as Cyclin D1 and c-Myc) that support OPC proliferation [179,180]. However, dysregulated activation of this pathway maintains OPCs in an undifferentiated state and represses genes required for maturation [128]. Pharmacological inhibition of Wnt signaling (e.g., XAV939) has been demonstrated to enhance oligodendrocyte differentiation and accelerate remyelination [181,182,183,184,185,186].

Another critical regulator is the Shh signaling pathway, which is particularly active during developmental myelination and regenerative responses [187]. The binding of Shh ligand to the PTCH1 receptor relieves inhibition of SMO, enabling activation of downstream Gli transcription factors (primarily Gli1), which drive transcriptional programs that promote OPC proliferation and survival [188]. Upregulation of Shh signaling has been associated with increased OPC recruitment in demyelinated lesions [189,190,191,192].

In contrast, the Notch signaling pathway mainly acts as a negative regulator of OPC differentiation (Table 1) [193,194]. Activation occurs through the interaction between the Notch1 receptor on OPCs and its ligand, Jagged1, expressed on neighboring cells such as astrocytes [195]. This engagement triggers γ-secretase-dependent cleavage of Notch1, releasing the NICD, which translocates to the nucleus and interacts with some transcriptional co-activators such as RBP-Jκ to induce the expression of some inhibitory genes like Hes5 [196]. Continuous Notch pathway signaling inhibits the differentiation of OPCs, thereby retaining their identity [193]. Pharmacological inhibition of this pathway employing γ-secretase inhibitors, like DAPT, has shown potential in promoting oligodendrocyte differentiation and remyelination by preventing Notch pathway activation [197,198].

**Table 1:** Table summarizing some therapeutic agents that promote OPC differentiation and remyelination in experimental models by modulating the Wnt/β-catenin, Shh, and Notch signaling pathways. Abbreviations: Shh (Sonic Hedgehog), DAPT (N-[N-(3,5-difluorophenacetyl)-L-alanyl]-S-phenylglycine t-butyl ester), and SMO (Smoothened).

Signaling Pathway	Compound	Target	Effects on Oligodendrocytes	References
Wnt/β-catenin	XAV939	Tankyrase (β-catenin)	Promotes OPC differentiation Anti-apoptotic effects on mature oligodendrocytes	[181,182,183,184,185]
	ICG-001	β-catenin	Promotes OPC differentiation	[186]
Shh	Purmorphamine	SMO		[189,190]
	Clobetasol			[191,192]
Notch	DAPT	γ-secretase		[197]
	MW167	γ-secretase		[198]

### 4.2. Enhancing Myelination via Neurotrophic Factors and Growth Molecules

Neurotrophic factors, such as BDNF, IGF-1, and NRG1 have demonstrated significant pro-myelinating effects by promoting oligodendrocyte survival, maturation, and myelin production in both developmental and regenerative contexts.

BDNF, primarily signaling through the TrkB receptor, activates several downstream pathways including the MAPK/ERK and PI3K/Akt cascades [199]. These signaling pathways converge to improve oligodendrocyte survival, differentiation, and myelin sheath formation [200]. BDNF-TrkB signaling has also been demonstrated to regulate local translation of myelin proteins like MBP in oligodendrocyte processes, which is critical for efficient and localized myelination (Table 2) [201].

IGF-1 exerts its effects through the IGF-1R, a receptor tyrosine kinase that activates the PI3K/Akt/mTOR axis, a central regulator of cell metabolism, growth, and protein synthesis [202]. IGF-1 drives OPC proliferation, survival, and transition to mature, myelinating oligodendrocytes [203]. Activation of mTORC1 by IGF-1 enhances lipid and protein biosynthesis required for myelin membrane production [204]. Pharmacological manipulation of this pathway has shown efficacy in promoting remyelination in some experimental demyelination models (Table 2) [205,206,207].

NRG1, particularly type III isoform, plays a pivotal role in axon–glia communication [208]. NRG1 type III is expressed on axonal membranes and interacts with ErbB2/ErbB3 receptor complexes on oligodendrocytes [209]. This interaction initiates intracellular signaling cascades, including PI3K/Akt and MAPK, which regulate myelin thickness and the timing of oligodendrocyte differentiation [210]. In those models where NRG1 expressions are dysregulated, exogenous administration of NRG1 or enhancement of its signaling has been shown to restore remyelination potential and improve axonal ensheathment, suggesting a therapeutic avenue for diseases such as MS (Table 2) [211,212].

**Table 2:** Table summarizing drugs that promote myelination through neurotrophic signaling pathways. Abbreviations: BDNF (brain-derived neurotrophic factor), 7,8-DHF (7,8-dihydroxyflavone), TrkB (tropomyosin receptor kinase B), IGF-1 (insulin-like growth factor 1), IGF-1R (insulin-like growth factor 1 receptor), mRNA (messenger ribonucleic acid), NRG1 (neuregulin 1), NRG1β1 (neuregulin 1 beta 1), ErbB4 (erythroblastic leukemia viral oncogene homolog 4), OPC (oligodendrocyte precursor cell), ErbB2 (erythroblastic leukemia viral oncogene homolog 2), CSPG (chondroitin sulfate proteoglycan), and IL-10 (interleukin 10).

Signaling Pathway	Compound	Target	Effects on Oligodendrocytes	References
BDNF/TrkB	7,8-DHF	TrkB	Reduces demyelination and axonal loss	[201]
IGF-1/ IGF-1R	IGF	IGF-1R	Reduces demyelination and upregulates mRNA encoding myelin proteins	[205,206]
			Induces remyelination	[207]
NRG1	NRG1β1	ErbB4	Inhibits OPC apoptosis in vitro	[211]
		Erb2/ErbB4	NRG-1 reduces CSPGs and increases IL-10 in those demyelinated areas	[212]

### 4.3. Epigenetic Modulation of Oligodendrocyte Fate

Epigenetic regulation is emerging as a potent mechanism in oligodendrocyte biology, influencing every stage of oligodendrocyte development from OPC proliferation to terminal differentiation and myelination [32]. Within the epigenetic regulators, HDACs have been shown to play key roles in OPC differentiation [213]. Pharmacological inhibition of HDACs plays a key role in regulating OPCs and their maturation via modulation of chromatin remodeling, thereby promoting transcriptional activation of those genes implicated in OPC proliferation, differentiation, and myelin gene expression [214]. Numerous small-molecule inhibitors have been implicated in the amelioration of MS symptoms by potentiating oligodendrocyte functionality (Table 3) [215,216,217,218].

Simultaneously, DNMTs take part in controlling oligodendrocyte development, maturation, and myelination by regulating DNA methylation dynamics throughout OPC differentiation [125]. In MS, dysregulated DNMT activity contributes to impaired oligodendrocyte differentiation, remyelination failure, and exacerbated neurodegeneration [125]. Some drugs improve the DNMT activity in MS preclinical models (Table 3) [219,220,221].

**Table 3:** Table summarizing HDAC inhibitors and DNMT activators that regulate OPC differentiation and promote remyelination in various experimental MS models. Abbreviations: HDAC (histone deacetylase), HDAC1 (histone deacetylase 1), HDAC2 (histone deacetylase 2), OPC (oligodendrocyte precursor cell), PI3K (phosphoinositide 3-kinase), DNMT (DNA methyltransferase), and GPR97 (G protein-coupled receptor 97).

Drug Family	Compound	Target	Effects on Oligodendrocytes	References
HDAC inhibitors	Valproic acid	HDAC1/2	Increases endogenous myelin repair by recruiting OPCs	[215]
			Promotes expression of associated myelin genes and oligodendrocyte function	[216]
	α-linolenic acid-valproic acid	HDAC1/2	Promote oligodendrocyte function	[217]
	LY294002	PI3K/HDAC inhibitor		[218]
DNMT activators	Curcumin	GPR97 agonist DNMT activator		[219,220]
	Vitamin C	DNMT activator		[221]

ncRNAs, primarily miRNAs, add an additional dimension to this complex regulatory network [222]. Advances in nanotechnology and gene therapy have facilitated the development of CNS-targeted delivery systems, such as nanoparticles and viral vectors, enabling efficient and specific in vivo delivery of miRNA-based therapeutics to modulate the oligodendrocyte epigenetic landscape and promote remyelination. Indeed, recent studies have demonstrated that the administration of a series of miRNA mimics, delivered with or without lentiviral vectors (e.g., miR-33-3p, miR-34c-5p, miR-124-5p, and miR-184), stimulated the differentiation of OPCs into mature oligodendrocytes in vitro [223,224].

### 4.4. Cell-Based Therapies and Transplantation Approaches

Cell transplantation therapies employing OPCs or oligodendrocytes derived from induced pluripotent stem cells (iPSC-OLs) represent a promising regenerative approach for demyelinating diseases, including MS [225,226]. These transplanted cells have shown the ability not only to differentiate into mature oligodendrocytes endowed with the capacity to remodel denuded axons, but also to provide essential trophic support through the secretion of a paracrine factor. These factors contribute to modulating the local microenvironment by promoting neuroprotection, enhancing survival and proliferation of endogenous cells, and reducing inflammatory responses, collectively facilitating a more favorable milieu for tissue repair and functional recovery [227,228].

Preclinical studies utilizing human embryonic stem cell-derived OPCs (hESC-OPCs) have provided compelling evidence for successful engraftment, migration, and functional remyelination in rodent models of MS. These studies underscore the therapeutic potential of stem cell-derived OPCs in restoring myelin integrity and improving electrophysiological outcomes [229,230,231,232,233].

To improve the survival, retention, and integration of those transplanted cells within the hostile environment of injured CNS tissue, biomaterial scaffolds and hydrogels have been employed [234,235]. These biomimetic materials replicate key physical and biochemical characteristics of the native extracellular matrix, providing mechanical support, modulating cell adhesion, and enhancing nutrient diffusion [236]. By creating a conducive microenvironment, these scaffolds enhance cell viability, promote OPC differentiation, and encourage axon-glial interactions essential for effective myelination [237].

Moreover, the advent of CRISPR/Cas9 genome-editing technology has opened new avenues for the generation of genetically engineered iPSCs with enhanced therapeutic potential [238,239]. Genome editing enables iPSCs to be programmed for the overexpression of those myelin-promoting factors that regulate oligodendrocyte differentiation and myelination [240]. This personalized cell therapy allows patient-specific enhancement of remyelination and functional recovery, while reducing immunogenicity risks [241].

### 4.5. Regulation of the Inflammatory Milieu

The inflammatory microenvironment within demyelinated lesions represents a major impediment to effective remyelination, particularly in the context of MS [20,21]. In the CNS, the sustained activation of immune cells alters the lesion milieu in a manner that is detrimental to oligodendrocyte function [26,27,28].

Several pharmacological interventions, concomitantly with the modulation of the inflammatory milieu, enable the differentiation of OPCs into mature oligodendrocytes [242]. Several agonists of PPARγ have demonstrated efficacy in dampening neuroinflammation and promoting OPCs differentiation [243]. Similarly, minocycline has been demonstrated to inhibit microglial activation and enhance remyelination outcomes [244]. The inhibition of CSF1R, which is essential for microglial survival and proliferation, has also been identified as a promising therapeutic approach to ameliorate the accumulation of pathogenic microglia and restore the lesion microenvironment in a manner that supports oligodendrocyte regeneration (Table 4) [245,246,247,248,249,250,251,252,253,254,255,256,257].

Importantly, targeting intracellular inflammasome pathways, mainly the NLRP3 inflammasome, has been recognized to mitigate chronic neuroinflammation and preserve oligodendrocyte populations [248]. The activation of the NLRP3 inflammasome promotes cleavage and subsequent release of the pro-inflammatory cytokines IL-1β and IL-18, thus amplifying tissue damage and exacerbating demyelination [249]. Pharmacological inhibitors of NLRP3 have demonstrated the ability to attenuate oligodendrocyte loss and promote remyelination in MS preclinical models, suggesting that inflammasome modulation may represent a viable avenue for therapeutic intervention [250].

**Table 4:** Table summarizing drugs that modulate PPARγ, CSF1R, and NLRP3 signaling pathways implicated in the inflammation linked to MS. Abbreviations: MS (Multiple Sclerosis), PPARγ (peroxisome proliferator-activated receptor gamma), OPC (oligodendrocyte precursor cell), CSF1R (colony-stimulating factor 1 receptor), CUP (cuprizone), FGFR (fibroblast growth factor receptor), VEGFR2 (vascular endothelial growth factor receptor 2), NLRP3 (NOD-, LRR- and pyrin domain-containing protein 3), and EAE (experimental autoimmune encephalomyelitis).

Compound	Activity	Effects on MS	References
Pioglitazone	PPARγ agonist	Promotes the conversion of OPCs into mature oligodendrocytes	[243]
Minocycline	Targets various microglial activation pathways	Allows remyelination by inhibiting microglial activity	[244]
PLX3397	CSF1R antagonist	Prevents demyelination, oligodendrocyte loss, and reactive astrocytosis induced by CUP treatment	[245]
		Enhances oligodendrocyte density and remyelination in CUP-treated mice	[246]
AZD4547	Blockade of FGFR, VEGFR2, and CSF1R	Increases the abundance of OPCs and mature oligodendrocytes in MS lesions	[247]
MCC950	Selective NLRP3 inflammasome inhibitor	Mitigates neuronal damage, demyelination, and oligodendrocyte loss in EAE mouse brains	[250]

### 4.6. Other Pharmacological Modulators of OPC Development and Myelin Repair

Several inhibitory molecules like Nogo-A and LINGO-1 negatively regulate OPC differentiation and remyelination through the activation of the RhoA/ROCK signaling pathway, which impairs cytoskeletal reorganization and the extension of cellular processes required for myelin repair [251,252]. Therapeutic blockade of Nogo-A and LINGO-1 alleviates this inhibition, promoting OPC maturation [253,254,255,256,257,258,259,260,261,262,263,264,265,266]. In parallel, some histamine antagonists modulate inflammatory microenvironments and may indirectly enhance oligodendrocyte survival and differentiation [257,258,259], while muscarinic M1 receptor antagonism directly affects OPC proliferation and differentiation through modulation of intracellular Ca^2+^ signaling pathways (Table 5) [260,261,262,263].

Thyroid hormone acts synergistically by upregulating myelin gene expression and promoting OPC maturation via nuclear thyroid hormone receptors, which regulate transcriptional programs essential for remyelination [264,265,266,267]. Semaphorin 4D (SEMA4D), a member of the axonal guidance cues, interacts with plexin receptors on oligodendrocytes, modulating both cytoskeletal dynamics and local inflammation [268]; its inhibition attenuates neuroinflammation and supports remyelination (Table 5) [269].

On the other hand, adrenergic signaling influences oligodendrocyte metabolism and survival under inflammatory conditions, presumably through the regulation of cAMP concentrations and subsequent PKA activation, which may interact with MAPK signaling pathways [270]. Finally, the MEK inhibitor trametinib regulates the MAPK/ERK signaling pathway, a key downstream mediator of growth factor and an extracellular signal integrator; suppression of aberrant MAPK activity facilitates the reestablishment of OPC differentiation and enhances myelin protein production (Table 5) [271].

**Table 5:** Table summarizing drugs that modulate alternative pathways involved in OPC differentiation and mature oligodendrocyte formation. Abbreviations: MS (multiple sclerosis), Nogo-A (neurite outgrowth inhibitor A), EAE (experimental autoimmune encephalomyelitis), LINGO-1 (leucine-rich repeat and immunoglobulin-like domain-containing Nogo receptor-interacting protein 1), CUP (cuprizone), H1 (histamine receptor 1), M1 (muscarinic acetylcholine receptor 1), M3 (muscarinic acetylcholine receptor 3), OPC (oligodendrocyte precursor cell), Sob-AM2 (sobetirome-AM2), SEMA4D (semaphorin 4D), α2 (alpha-2 adrenergic receptor), and MEK (mitogen-activated protein kinase kinase).

Compound	Activity	Effects on MS	References
Anti-Nogo-A	Nogo-A blockade	Improves remyelination in EAE preclinical MS model	[253]
Anti-LINGO-1	LINGO-1 blockade	Improves remyelination in EAE preclinical MS model	[254]
		Improves remyelination in CUP-induced demyelination	[255]
		Opicinumab improves remyelination	[256]
Clemastine	H1 antagonist M1/M3 antagonist	Promotes oligodendrocyte function in EAE preclinical MS model	[258,259]
PIPE-307	M1 antagonists		[262]
PIPE-791			[263]
Thyroid hormone	TR agonists	Activates OPCs and enables remyelination in EAE model	[265]
TG68/IS25		Induce OPCs differentiation and maturation in vitro	[266]
Sobetirome/Sob-AM2		Activates OPCs and enables remyelination in EAE model	[267]
Anti-SEMA4D	SEMA4D blockade	Reduces apoptosis of OPCs and promotes their differentiation in vitro	[269]
Guanabenz	α2 adrenergic receptor agonist	Enhances oligodendrocyte survival in vitro	[270]
Trametinib	MEK inhibitor	Promotes remyelination and increases the formation of mature oligodendrocytes in the EAE preclinical MS model	[271]

## 5. Conclusions

Oligodendrocytes represent a cornerstone in the pathophysiology of MS, functioning as key cellular mediators of both demyelination and remyelination processes within the CNS. The selective vulnerability of oligodendrocytes to immune-mediated attacks, oxidative stress, and mitochondrial dysfunction concludes with the disruption of myelin integrity, which is pivotal for axonal conduction and neural functionality. This disease initiates a cascade of secondary neurodegenerative events, including axonal transection and neuronal loss, which correlate strongly with the progressive disability observed clinically in MS patients. The heterogeneity of OPCs and their variable capacity to respond to demyelinating insults is an emerging area of interest. OPCs possess intrinsic potential for proliferation, migration, and differentiation to replace lost oligodendrocytes; however, within chronic MS lesions, remyelination frequently fails due to a hostile extracellular environment, sustained inflammatory activity, and dysregulated glial–immune cell interactions.

Moreover, current therapeutic strategies have mainly targeted the modulation of aberrant immune responses to reduce relapse rates and slow disease progression. While immunomodulatory treatments have proven effective in managing the inflammatory phase of MS, they do not adequately address the critical need for myelin repair. Recent advances in experimental models have provided insight on some remyelination-enhancing agents, such as small molecules, mAbs, and cell-based therapies, which aim to stimulate endogenous OPCs or provide exogenous sources of myelinating cells. These interventions hold potential to restore functional myelin sheaths and preserve axonal integrity.

However, translating these findings into clinical practice remains a considerable challenge, as the interplay between inflammation, neurodegeneration, and repair mechanisms requires a nuanced therapeutic approach. The demyelinated environment presents a significant barrier to remyelination, involving not only immune cells but also astrocytes and microglia, which can exert supportive and detrimental effects on oligodendrocyte biology. Thus, an integrative understanding of the CNS microenvironment is essential to develop combination therapies that simultaneously target inflammation, promote remyelination, and support neuronal survival.

Future research should also focus on identifying reliable biomarkers that reflect oligodendrocyte health and remyelination status, which would greatly enhance the ability to monitor disease progression and therapeutic efficacy in vivo. Advances in neuroimaging techniques, such as myelin-sensitive MRI sequences, alongside molecular biomarkers detectable in several fluids (such as CSF or blood), may offer non-invasive means to track repair processes and tailor individualized treatment strategies.

In summary, the complexity of MS pathogenesis underscores the necessity of a multi-pronged approach that exceeds traditional immune suppression to integrate regenerative medicine and neuroprotection. By deepening our understanding of oligodendrocyte biology and the pathological mechanisms impairing myelin repair, the scientific community is making progress toward the development of transformative therapies that not only attenuate disease activity but also promote restoration of CNS integrity. Such progress presents the potential to enhance long-term outcomes and quality of life for patients affected by this disorder, representing a paradigm shift from symptomatic management to genuine disease modification and CNS repair.

## Data Availability

Not applicable. No new data were generated.

## Abbreviations

The following abbreviations are used in this manuscript:

OH	Hydroxyl radical
8-OHdG	8-hydroxy-2′-deoxyguanosine
ADCC	Antibody-dependent cellular cytotoxicity
ADP	Adenosine diphosphate
aFn	Aggregated fibronectin
Akt	Protein kinase B (PKB)
AMPA	α-amino-3-hydroxy-5-methyl-4-isoxazolepropionic acid
APC	Antigen-presenting cell
ATF6	Activating transcription factor 6
ATP	Adenosine triphosphate
BBB	Blood–brain barrier
BCR	B cell receptor
BDNF	Brain-derived neurotrophic factor
bHLH	Basic helix-loop-helix
BiP	Binding immunoglobulin protein
BMP	Bone morphogenetic protein
BMP4	Bone morphogenetic protein 4
Breg	Regulatory B cell
C1q	Complement component 1q
C3	Complement component 3
C3b	Complement component 3b
C5b-9	Complement components 5b to 9
Ca^2+^	Calcium ion
CCL2	C-C motif chemokine ligand 2
CCL20	C-C motif chemokine ligand 20
CCL5	C-C motif chemokine ligand 5
CD28	Cluster of differentiation 28
CD4	Cluster of differentiation 4
CD44	Cluster of differentiation 44
CD8	Cluster of differentiation 8
CD80	Cluster of differentiation 80
CD86	Cluster of differentiation 86
CIITA	Class II major histocompatibility complex transactivator
CNS	Central nervous system
CSF	Cerebrospinal fluid
CSF1R	Colony stimulating factor 1 receptor
CSPG	Chondroitin sulfate proteoglycan
CUP	Cuprizone
CXCL1	C-X-C motif chemokine ligand 1
CXCL10	C-X-C motif chemokine ligand 10
CXCL12	C-X-C motif chemokine ligand 12
CXCL2	C-X-C motif chemokine ligand 2
DAMP	Damage-associated molecular pattern
DAPT	N-[N-(3,5-difluorophenacetyl)-L-alanyl]-S-phenylglycine t-butyl ester
DC	Dendritic cell
DISC	Death-inducing signaling complex
DMT	Disease-modifying therapy
DNA	Deoxyribonucleic acid
DNMT	DNA methyltransferase
EAE	Experimental autoimmune encephalomyelitis
EBV	Epstein–Barr virus
eIF2B	Eukaryotic initiation factor 2B
eIF2α	Eukaryotic initiation factor 2 alpha
ER	Endoplasmic reticulum
ERAD	Endoplasmic reticulum-associated degradation
ErbB2	Erythroblastic leukemia viral oncogene homolog 2
ErbB3	Erythroblastic leukemia viral oncogene homolog 3
ErbB4	Erythroblastic leukemia viral oncogene homolog 4
ERK	Extracellular signal-regulated kinase
ETC	Electron transport chain
EZH2	Enhancer of zeste homolog 2
FADD	Fas-associated protein with death domain
FADH_2_	Flavin adenine dinucleotide (reduced form)
Fas	Fas receptor
FasL	Fas ligand
Fcγ	Fc gamma
Fe	Iron
Fe^2+^	Ferrous iron
FGF-2	Fibroblast growth factor 2
FGFR	Fibroblast growth factor receptor
GFAP	Glial fibrillary acidic protein
Gli1	GLI family zinc finger 1
GPR97	G protein-coupled receptor 97
GRP78	78 kDa glucose-regulated protein
GRP94	94 kDa glucose-regulated protein
H^+^	Proton
H1	Histamine receptor 1
H_2_O_2_	Hydrogen peroxide
H3K27me3	Trimethylation of lysine 27 on histone H3
HDAC	Histone deacetylase
HDAC1	Histone deacetylase 1
HDAC2	Histone deacetylase 2
Hes1	Hairy and enhancer of split 1
Hes5	Hairy and enhancer of split 5
hESC-OPC	Human embryonic stem cell-oligodendrocyte precursor cell
ICAM-1	Intercellular adhesion molecule 1
ID2	Inhibitor of DNA binding 2
ID4	Inhibitor of DNA binding 4
IFN-γ	Interferon gamma
IGF-1	Insulin-like growth factor 1
IGF-1R	Insulin-like growth factor 1 receptor
IgG	Immunoglobulin G
IL-10	Interleukin 10
IL-12	Interleukin 12
IL-17	Interleukin 17
IL-17A	Interleukin 17A
IL-17F	Interleukin 17F
IL-1β	Interleukin 1 beta
IL-21	Interleukin 21
IL-22	Interleukin 22
IL-23	Interleukin 23
IL-33	Interleukin 33
IL-6	Interleukin 6
iNOS	Inducible nitric oxide synthase
iPSC-OL	Induced pluripotent stem cell-oligodendrocyte
IRE1α	Inositol requiring enzyme 1 alpha
IRF3	Interferon regulatory factor 3
ISR	Integrated stress response
JAM	Junctional adhesion molecule
K^+^	Potassium ion
LAR	Leukocyte common antigen-related receptor
LFA-1	Lymphocyte function-associated antigen 1
LINGO-1	Leucine-rich repeat and immunoglobulin-like domain-containing Nogo receptor-interacting protein 1
LRP5/6	Low-density lipoprotein receptor-related protein 5/6
LT-α	Lymphotoxin alpha
M1	Muscarinic acetylcholine receptor 1
M3	Muscarinic acetylcholine receptor 3
mAb	Monoclonal antibody
MAC	Membrane attack complex
MAG	Myelin-associated glycoprotein
MAPK	Mitogen-activated protein kinase
MBP	Myelin basic protein
MEK	Mitogen-activated protein kinase kinase
MHC-I	Major histocompatibility complex class I
MHC-II	Major histocompatibility complex class II
miRNA	MicroRNA
MLKL	Mixed lineage kinase domain-like protein
MMP	Matrix metalloproteinase
MnSOD	Manganese superoxide dismutase
MOG	Myelin oligodendrocyte glycoprotein
MOMP	Mitochondrial outer membrane permeabilization
mRNA	Messenger ribonucleic acid
MS	Multiple sclerosis
mtDNA	Mitochondrial DNA
mTOR	Mechanistic target of rapamycin
mTOR1	Mechanistic target of rapamycin complex 1
MyD88	Myeloid differentiation primary response 88
MYRF	Myelin regulatory factor
Na^+^	Sodium ion
Na^+^/K^+^-ATPase	Sodium/potassium-ATPase
NADH	Nicotinamide adenine dinucleotide (reduced form)
NADPH	Nicotinamide adenine dinucleotide phosphate (reduced form)
NF-κB	Nuclear factor kappa-light-chain-enhancer of activated B cells
NICD	Notch intracellular domain
NK	Natural killer
NKX2.2	NK2 homeobox 2
NLRP3	NOD-, LRR- and pyrin domain-containing protein 3
NO	Nitric oxide
Nogo-A	Neurite outgrowth inhibitor A
NOX2	NADPH oxidase 2
NRG1	Neuregulin 1
NRG1β1	Neuregulin 1 beta 1
O_2_	Molecular oxygen
O_2_^−^	Superoxide anion
OLIG1	Oligodendrocyte transcription factor 1
OLIG2	Oligodendrocyte transcription factor 2
ONOO^−^	Peroxynitrite
OPC	Oligodendrocyte progenitor cell
OXPHOS	Oxidative phosphorylation
PAMP	Pathogen-associated molecular pattern
PDGF-A	Platelet-derived growth factor A
PECAM-1	Platelet endothelial cell adhesion molecule 1
PERK	Protein kinase R (PKR)-like endoplasmic reticulum kinase
PI3K	Phosphoinositide 3-kinase
PLP	Proteolipid protein
PLP1	Proteolipid protein 1
PPARγ	Peroxisome proliferator-activated receptor gamma
PPMS	Primary-progressive multiple sclerosis
PRMS	Progressive-relapsing multiple sclerosis
PRC2	Polycomb repressive complex 2
PRR	Pattern recognition receptor
PTCH1	Patched 1
PTPσ	Protein tyrosine phosphatase sigma
RAGE	Receptor for advanced glycation endproduct
RBP-Jκ	Recombination signal binding protein for immunoglobulin kappa J region
RhoA	RhoA GTPase
RIPK1	Receptor-interacting protein kinase 1
RIPK3	Receptor-interacting protein kinase 3
RNS	Reactive nitrogen species
ROCK	Rho-associated coiled-coil containing protein kinase
ROS	Reactive oxygen species
RRMS	Relapsing-remitting multiple sclerosis
S100β	S100 protein beta
SEMA4D	Semaphorin 4D
Shh	Sonic hedgehog
SMO	Smoothened
Sob-AM2	Sobetirome-AM2
SOX10	SRY-box transcription factor 10
Sox6	SRY-box transcription factor 6
SPMS	Secondary-progressive multiple sclerosis
STAT1	Signal transducer and activator of transcription 1
STAT3	Signal transducer and activator of transcription 3
sTNF	Soluble tumor necrosis factor
TCF7L2	Transcription factor 7 like 2
TCR	T cell receptor
Tfh	T follicular helper
TGF-β	Transforming growth factor beta
TLR	Toll-like receptor
TNFR1	Tumor necrosis factor receptor 1
TNF-α	Tumor necrosis factor alpha
TRIF	TIR-domain-containing adapter-inducing interferon beta
TrkB	Tropomyosin receptor kinase B
UPR	Unfolded protein response
VCAM-1	Vascular cell adhesion molecule 1
VEGFR2	Vascular endothelial growth factor receptor 2
VLA-4	Very late antigen 4
Wnt	Wingless/integrated 1
XBP1	X-box binding protein 1
ZO-1	Zonula occludens 1
α2	Alpha 2 adrenergic receptor

## References

[1] Haki M., Al-Biati H.A., Al-Tameemi Z.S., Ali I.S., Al-Hussaniy H.A. (2024). Review of multiple sclerosis: Epidemiology, etiology, pathophysiology, and treatment. Medicine.

[2] Coyle P.K. (2021). What Can We Learn from Sex Differences in MS?. J. Pers. Med..

[3] Prosperini L., Lucchini M., Ruggieri S., Tortorella C., Haggiag S., Mirabella M., Pozzilli C., Gasperini C. (2022). Shift of multiple sclerosis onset towards older age. J. Neurol. Neurosurg. Psychiatry.

[4] Sabel C.E., Pearson J.F., Mason D.F., Willoughby E., Abernethy D.A., Taylor B.V. (2021). The latitude gradient for multiple sclerosis prevalence is established in the early life course. Brain.

[5] Olsson T., Barcellos L.F., Alfredsson L. (2017). Interactions between genetic, lifestyle and environmental risk factors for multiple sclerosis. Nat. Rev. Neurol..

[6] Waubant E., Lucas R., Mowry E., Graves J., Olsson T., Alfredsson L., Langer-Gould A. (2019). Environmental and genetic risk factors for MS: An integrated review. Ann. Clin. Transl. Neurol..

[7] Oudejans E., Luchicchi A., Strijbis E.M.M., Geurts J.J.G., van Dam A.M. (2020). Is MS affecting the CNS only? Lessons from clinic to myelin pathophysiology. Neurol. Neuroimmunol. Neuroinflamm..

[8] Stavropoulou De Lorenzo S., Bakirtzis C., Konstantinidou N., Kesidou E., Parissis D., Evangelopoulos M.E., Elsayed D., Hamdy E., Said S., Grigoriadis N. (2023). How Early Is Early Multiple Sclerosis?. J. Clin. Med..

[9] Portaccio E., Magyari M., Havrdova E.K., Ruet A., Brochet B., Scalfari A., Di Filippo M., Tur C., Montalban X., Amato M.P. (2024). Multiple sclerosis: Emerging epidemiological trends and redefining the clinical course. Lancet Reg. Health Eur..

[10] Benedict R.H.B., Amato M.P., DeLuca J., Geurts J.J.G. (2020). Cognitive impairment in multiple sclerosis: Clinical management, MRI, and therapeutic avenues. Lancet Neurol..

[11] Pintér A., Cseh D., Sárközi A., Illigens B.M., Siepmann T. (2015). Autonomic Dysregulation in Multiple Sclerosis. Int. J. Mol. Sci..

[12] Liu Z., Liao Q., Wen H., Zhang Y. (2021). Disease modifying therapies in relapsing-remitting multiple sclerosis: A systematic review and network meta-analysis. Autoimmun. Rev..

[13] Ziemssen T., Bhan V., Chataway J., Chitnis T., Campbell Cree B.A., Havrdova E.K., Kappos L., Labauge P., Miller A., Nakahara J. (2022). Secondary Progressive Multiple Sclerosis: A Review of Clinical Characteristics, Definition, Prognostic Tools, and Disease-Modifying Therapies. Neurol. Neuroimmunol. Neuroinflamm..

[14] Sempik I., Dziadkowiak E., Moreira H., Zimny A., Pokryszko-Dragan A. (2024). Primary Progressive Multiple Sclerosis-A Key to Understanding and Managing Disease Progression. Int. J. Mol. Sci..

[15] Tullman M.J., Oshinsky R.J., Lublin F.D., Cutter G.R. (2004). Clinical characteristics of progressive relapsing multiple sclerosis. Mult. Scler..

[16] Arredondo-Robles A.V., Rodríguez-López K.P., Ávila-Avilés R.D. (2025). Clinical Management in Multiple Sclerosis. Neuroglia.

[17] Huang W.J., Chen W.W., Zhang X. (2017). Multiple sclerosis: Pathology, diagnosis and treatments. Exp. Ther. Med..

[18] Multz R.A., Jamshidi P., Ahrendsen J.T. (2025). Multiple sclerosis: A practical review for pathologists. J. Pathol. Transl. Med..

[19] Balasa R., Barcutean L., Mosora O., Manu D. (2021). Reviewing the Significance of Blood–Brain Barrier Disruption in Multiple Sclerosis Pathology and Treatment. Int. J. Mol. Sci..

[20] Zierfuss B., Larochelle C., Prat A. (2024). Blood–brain barrier dysfunction in multiple sclerosis: Causes, consequences, and potential effects of therapies. Lancet Neurol..

[21] Suknjaja V., Sakalaš L., Ilin M. (2024). EARLY ONSET OF MULTIPLE SCLEROSIS–CLINICAL FEATURES. Acta Clin. Croat..

[22] Murtonen A., Lehto J.T., Sumelahti M.L. (2021). End of life in multiple sclerosis: Disability, causes and place of death among cases diagnosed from 1981 to 2010 in Pirkanmaa hospital district in Western Finland. Mult. Scler. Relat. Disord..

[23] Miyata S., Wake H. (2024). Editorial: Oligodendrocytes: From their development to function and dysfunction. Front. Cell Neurosci..

[24] Wellman S.M., Cambi F., Kozai T.D. (2018). The role of oligodendrocytes and their progenitors on neural interface technology: A novel perspective on tissue regeneration and repair. Biomaterials.

[25] Nave K.A., Asadollahi E., Sasmita A. (2023). Expanding the function of oligodendrocytes to brain energy metabolism. Curr. Opin. Neurobiol..

[26] Meyer-Arndt L., Kerkering J., Kuehl T., Infante A.G., Paul F., Rosiewicz K.S., Siffrin V., Alisch M. (2023). Inflammatory Cytokines Associated with Multiple Sclerosis Directly Induce Alterations of Neuronal Cytoarchitecture in Human Neurons. J. Neuroimmune Pharmacol..

[27] Silva B.D., Viero F.T., Rodrigues P., Trevisan G. (2024). Nitric oxide involvement in the disability and active disease of multiple sclerosis: Systematic review and meta-analysis. Nitric Oxide.

[28] Piñar-Morales R., Durán R., Bautista-García A., García-Mansilla M.J., Aliaga-Gaspar P., Vives-Montero F., Barrero-Hernández F.J. (2025). The impact of oxidative stress on symptoms associated with multiple sclerosis. Sci. Rep..

[29] Lulu S., Waubant E. (2013). Humoral-targeted immunotherapies in multiple sclerosis. Neurotherapeutics.

[30] Akay L.A., Effenberger A.H., Tsai L.H. (2021). Cell of all trades: Oligodendrocyte precursor cells in synaptic, vascular, and immune function. Genes. Dev..

[31] Tepavčević V., Lubetzki C. (2022). Oligodendrocyte progenitor cell recruitment and remyelination in multiple sclerosis: The more, the merrier?. Brain.

[32] Ma Q., Shams H., Didonna A., Baranzini S.E., Cree B.A.C., Hauser S.L., Henry R.G., Oksenberg J.R. (2023). Integration of epigenetic and genetic profiles identifies multiple sclerosis disease-critical cell types and genes. Commun. Biol..

[33] Koutsoudaki P.N., Papadopoulos D., Passias P.G., Koutsoudaki P., Gorgoulis V.G. (2020). Cellular senescence and failure of myelin repair in multiple sclerosis. Mech. Ageing Dev..

[34] Jäkel S., Agirre E., Mendanha Falcão A., van Bruggen D., Lee K.W., Knuesel I., Malhotra D., Ffrench-Constant C., Williams A., Castelo-Branco G. (2019). Altered human oligodendrocyte heterogeneity in multiple sclerosis. Nature.

[35] Zveik O., Rechtman A., Ganz T., Vaknin-Dembinsky A. (2024). The interplay of inflammation and remyelination: Rethinking MS treatment with a focus on oligodendrocyte progenitor cells. Mol. Neurodegener..

[36] You Y., Gupta V. (2018). The Extracellular Matrix and Remyelination Strategies in Multiple Sclerosis. eNeuro.

[37] Gaesser J.M., Fyffe-Maricich S.L. (2016). Intracellular signaling pathway regulation of myelination and remyelination in the CNS. Exp. Neurol..

[38] Simkins T.J., Duncan G.J., Bourdette D. (2021). Chronic Demyelination and Axonal Degeneration in Multiple Sclerosis: Pathogenesis and Therapeutic Implications. Curr. Neurol. Neurosci. Rep..

[39] Looser Z.J., Faik Z., Ravotto L., Zanker H.S., Jung R.B., Werner H.B., Ruhwedel T., Möbius W., Bergles D.E., Barros L.F. (2024). Oligodendrocyte-axon metabolic coupling is mediated by extracellular K^+^ and maintains axonal health. Nat. Neurosci..

[40] Li S., Sheng Z.H. (2023). Oligodendrocyte-derived transcellular signaling regulates axonal energy metabolism. Curr. Opin. Neurobiol..

[41] Schäffner E., Bosch-Queralt M., Edgar J.M., Lehning M., Strauß J., Fleischer N., Kungl T., Wieghofer P., Berghoff S.A., Reinert T. (2023). Myelin insulation as a risk factor for axonal degeneration in autoimmune demyelinating disease. Nat. Neurosci..

[42] Wu X., Wang S., Xue T., Tan X., Li J., Chen Z., Wang Z. (2024). Disease-modifying therapy in progressive multiple sclerosis: A systematic review and network meta-analysis of randomized controlled trials. Front. Neurol..

[43] Plemel J.R., Liu W.Q., Yong V.W. (2017). Remyelination therapies: A new direction and challenge in multiple sclerosis. Nat. Rev. Drug Discov..

[44] Sharma T., Mehan S., Tiwari A., Khan Z., Gupta G.D., Narula A.S. (2025). Targeting Oligodendrocyte Dynamics and Remyelination: Emerging Therapies and Personalized Approaches in Multiple Sclerosis Management. Curr. Neurovasc. Res..

[45] Manousi A., Göttle P., Reiche L., Cui Q.L., Healy L.M., Akkermann R., Gruchot J., Schira-Heinen J., Antel J.P., Hartung H.P. (2021). Identification of novel myelin repair drugs by modulation of oligodendroglial differentiation competence. eBioMedicine.

[46] Medina-Rodríguez E.M., Bribián A., Boyd A., Palomo V., Pastor J., Lagares A., Gil C., Martínez A., Williams A., de Castro F. (2017). Promoting in vivo remyelination with small molecules: A neuroreparative pharmacological treatment for Multiple Sclerosis. Sci. Rep..

[47] Christodoulou M.V., Petkou E., Atzemoglou N., Gkorla E., Karamitrou A., Simos Y.V., Bellos S., Bekiari C., Kouklis P., Konitsiotis S. (2024). Cell replacement therapy with stem cells in multiple sclerosis, a systematic review. Hum. Cell.

[48] Paroni M., Maltese V., De Simone M., Ranzani V., Larghi P., Fenoglio C., Pietroboni A.M., De Riz M.A., Crosti M.C., Maglie S. (2017). Recognition of viral and self-antigens by T_H_1 and T_H_1/T_H_17 central memory cells in patients with multiple sclerosis reveals distinct roles in immune surveillance and relapses. J. Allergy Clin. Immunol..

[49] Lai S., Wu X., Liu Y., Liu B., Wu H., Ma K. (2024). Interaction between Th17 and central nervous system in multiple sclerosis. Brain Behav. Immun. Health.

[50] Chastain E.M., Duncan D.S., Rodgers J.M., Miller S.D. (2011). The role of antigen presenting cells in multiple sclerosis. Biochim. Biophys. Acta.

[51] Sonar S.A., Lal G. (2017). Differentiation and Transmigration of CD4 T Cells in Neuroinflammation and Autoimmunity. Front. Immunol..

[52] Li R., Li H., Yang X., Hu H., Liu P., Liu H. (2022). Crosstalk between dendritic cells and regulatory T cells: Protective effect and therapeutic potential in multiple sclerosis. Front. Immunol..

[53] Ma M., Jiang W., Zhou R. (2024). DAMPs and DAMP-sensing receptors in inflammation and diseases. Immunity.

[54] Lassmann H., Ransohoff R.M. (2004). The CD4-Th1 model for multiple sclerosis: A critical re-appraisal. Trends Immunol..

[55] Catenacci R.B., Galleguillos D., Rhodes A., Phillips S., Calabresi P.A. (2025). MHC class I and II expression and induction in oligodendrocytes varies with age. Sci. Rep..

[56] Moser T., Akgün K., Proschmann U., Sellner J., Ziemssen T. (2020). The role of TH17 cells in multiple sclerosis: Therapeutic implications. Autoimmun. Rev..

[57] Milovanovic J., Arsenijevic A., Stojanovic B., Kanjevac T., Arsenijevic D., Radosavljevic G., Milovanovic M., Arsenijevic N. (2020). Interleukin-17 in Chronic Inflammatory Neurological Diseases. Front. Immunol..

[58] Setiadi A.F., Abbas A.R., Jeet S., Wong K., Bischof A., Peng I., Lee J., Bremer M., Eggers E.L., DeVoss J. (2019). IL-17A is associated with the breakdown of the blood-brain barrier in relapsing-remitting multiple sclerosis. J. Neuroimmunol..

[59] Behrens M., Comabella M., Lünemann J.D. (2024). EBV-specific T-cell immunity: Relevance for multiple sclerosis. Front. Immunol..

[60] Thomas O.G., Olsson T. (2023). Mimicking the brain: Epstein-Barr virus and foreign agents as drivers of neuroimmune attack in multiple sclerosis. Front. Immunol..

[61] Comi G., Bar-Or A., Lassmann H., Uccelli A., Hartung H.P., Montalban X., Sørensen P.S., Hohlfeld R., Hauser S.L. (2021). Expert Panel of the 27th Annual Meeting of the European Charcot Foundation. Role of B Cells in Multiple Sclerosis and Related Disorders. Ann. Neurol..

[62] Wekerle H. (2017). B cells in multiple sclerosis. Autoimmunity.

[63] Arneth B.M. (2019). Impact of B cells to the pathophysiology of multiple sclerosis. J. Neuroinflamm..

[64] Negron A., Stüve O., Forsthuber T.G. (2020). Ectopic Lymphoid Follicles in Multiple Sclerosis: Centers for Disease Control?. Front. Neurol..

[65] Bonnan M. (2015). Intrathecal IgG synthesis: A resistant and valuable target for future multiple sclerosis treatments. Mult. Scler. Int..

[66] Saez-Calveras N., Stuve O. (2022). The role of the complement system in Multiple Sclerosis: A review. Front. Immunol..

[67] Ingram G., Loveless S., Howell O.W., Hakobyan S., Dancey B., Harris C.L., Robertson N.P., Neal J.W., Morgan B.P. (2014). Complement activation in multiple sclerosis plaques: An immunohistochemical analysis. Acta Neuropathol. Commun..

[68] Morille J., Mandon M., Rodriguez S., Roulois D., Leonard S., Garcia A., Wiertlewski S., Le Page E., Berthelot L., Nicot A. (2022). Multiple Sclerosis CSF Is Enriched With Follicular T Cells Displaying a Th1/Eomes Signature. Neurol. Neuroimmunol. Neuroinflamm..

[69] Jones B.E., Maerz M.D., Buckner J.H. (2018). IL-6: A cytokine at the crossroads of autoimmunity. Curr. Opin. Immunol..

[70] Varghese J.F., Kaskow B.J., von Glehn F., Case J., Li Z., Julé A.M., Berdan E., Ho Sui S.J., Hu Y., Krishnan R. (2024). Human regulatory memory B cells defined by expression of TIM-1 and TIGIT are dysfunctional in multiple sclerosis. Front. Immunol..

[71] Alahmari A. (2021). Blood-Brain Barrier Overview: Structural and Functional Correlation. Neural Plast..

[72] Cannella B., Raine C.S. (1995). The adhesion molecule and cytokine profile of multiple sclerosis lesions. Ann. Neurol..

[73] Elovaara I., Ukkonen M., Leppäkynnäs M., Lehtimäki T., Luomala M., Peltola J., Dastidar P. (2000). Adhesion molecules in multiple sclerosis: Relation to subtypes of disease and methylprednisolone therapy. Arch. Neurol..

[74] Schwab N., Schneider-Hohendorf T., Wiendl H. (2015). Therapeutic uses of anti-α4-integrin (anti-VLA-4) antibodies in multiple sclerosis. Int. Immunol..

[75] Bö L., Peterson J.W., Mørk S., Hoffman P.A., Gallatin W.M., Ransohoff R.M., Trapp B.D. (1996). Distribution of immunoglobulin superfamily members ICAM-1, -2, -3, and the beta 2 integrin LFA-1 in multiple sclerosis lesions. J. Neuropathol. Exp. Neurol..

[76] Yang J., Ran M., Li H., Lin Y., Ma K., Yang Y., Fu X., Yang S. (2022). New insight into neurological degeneration: Inflammatory cytokines and blood-brain barrier. Front. Mol. Neurosci..

[77] Larochelle C., Alvarez J.I., Prat A. (2011). How do immune cells overcome the blood-brain barrier in multiple sclerosis?. FEBS Lett..

[78] Cui L.Y., Chu S.F., Chen N.H. (2020). The role of chemokines and chemokine receptors in multiple sclerosis. Int. Immunopharmacol..

[79] Wimmer I., Tietz S., Nishihara H., Deutsch U., Sallusto F., Gosselet F., Lyck R., Muller W.A., Lassmann H., Engelhardt B. (2019). PECAM-1 Stabilizes Blood-Brain Barrier Integrity and Favors Paracellular T-Cell Diapedesis Across the Blood-Brain Barrier During Neuroinflammation. Front. Immunol..

[80] Tietz S., Périnat T., Greene G., Enzmann G., Deutsch U., Adams R., Imhof B., Aurrand-Lions M., Engelhardt B. (2018). Lack of junctional adhesion molecule (JAM)-B ameliorates experimental autoimmune encephalomyelitis. Brain Behav. Immun..

[81] Kim S., Jung U.J., Kim S.R. (2024). Role of Oxidative Stress in Blood-Brain Barrier Disruption and Neurodegenerative Diseases. Antioxidants.

[82] Sun Y., Yu H., Guan Y. (2023). Glia Connect Inflammation and Neurodegeneration in Multiple Sclerosis. Neurosci. Bull..

[83] Zhang X., Chen F., Sun M., Wu N., Liu B., Yi X., Ge R., Fan X. (2023). Microglia in the context of multiple sclerosis. Front. Neurol..

[84] Asadzadeh Manjili F., Yousefi-Ahmadipour A., Kazemi Arababadi M. (2020). The roles played by TLR4 in the pathogenesis of multiple sclerosis; A systematic review article. Immunol. Lett..

[85] Fan H., Fu Q., Du G., Qin L., Shi X., Wang D., Yang Y. (2024). Microglial Mayhem NLRP3 Inflammasome’s Role in Multiple Sclerosis Pathology. CNS Neurosci. Ther..

[86] Zheng C., Chen J., Chu F., Zhu J., Jin T. (2020). Inflammatory Role of TLR-MyD88 Signaling in Multiple Sclerosis. Front. Mol. Neurosci..

[87] Timmerman R., Zuiderwijk-Sick E.A., Bajramovic J.J. (2022). P2Y6 receptor-mediated signaling amplifies TLR-induced pro-inflammatory responses in microglia. Front. Immunol..

[88] di Penta A., Moreno B., Reix S., Fernandez-Diez B., Villanueva M., Errea O., Escala N., Vandenbroeck K., Comella J.X., Villoslada P. (2013). Oxidative stress and proinflammatory cytokines contribute to demyelination and axonal damage in a cerebellar culture model of neuroinflammation. PLoS ONE.

[89] Smith A.N., Shaughness M., Collier S., Hopkins D., Byrnes K.R. (2022). Therapeutic targeting of microglia mediated oxidative stress after neurotrauma. Front. Med..

[90] Edison P. (2024). Astroglial activation: Current concepts and future directions. Alzheimers Dement..

[91] García-Domínguez M. (2025). Relationship of S100 Proteins with Neuroinflammation. Biomolecules.

[92] Rao X., Hua F., Zhang L., Lin Y., Fang P., Chen S., Ying J., Wang X. (2022). Dual roles of interleukin-33 in cognitive function by regulating central nervous system inflammation. J. Transl. Med..

[93] Wiese S., Karus M., Faissner A. (2012). Astrocytes as a source for extracellular matrix molecules and cytokines. Front. Pharmacol..

[94] Blake M.R., Gardner R.T., Jin H., Staffenson M.A., Rueb N.J., Barrios A.M., Dudley G.B., Cohen M.S., Habecker B.A. (2022). Small Molecules Targeting PTPσ-Trk Interactions Promote Sympathetic Nerve Regeneration. ACS Chem. Neurosci..

[95] Hosseini S.M., Alizadeh A., Shahsavani N., Chopek J., Ahlfors J.E., Karimi-Abdolrezaee S. (2022). Suppressing CSPG/LAR/PTPσ Axis Facilitates Neuronal Replacement and Synaptogenesis by Human Neural Precursor Grafts and Improves Recovery after Spinal Cord Injury. J. Neurosci..

[96] Domingues H.S., Portugal C.C., Socodato R., Relvas J.B. (2016). Oligodendrocyte, Astrocyte, and Microglia Crosstalk in Myelin Development, Damage, and Repair. Front. Cell Dev. Biol..

[97] Miron V.E., Franklin R.J. (2014). Macrophages and CNS remyelination. J. Neurochem..

[98] Lei Z., Lin W. (2024). Mechanisms Governing Oligodendrocyte Viability in Multiple Sclerosis and Its Animal Models. Cells.

[99] Denic A., Wootla B., Rodriguez M. (2013). CD8^+^ T cells in multiple sclerosis. Expert. Opin. Ther. Targets.

[100] Saxena A., Bauer J., Scheikl T., Zappulla J., Audebert M., Desbois S., Waisman A., Lassmann H., Liblau R.S., Mars L.T. (2008). Cutting edge: Multiple sclerosis-like lesions induced by effector CD8 T cells recognizing a sequestered antigen on oligodendrocytes. J. Immunol..

[101] Traka M., Podojil J.R., McCarthy D.P., Miller S.D., Popko B. (2016). Oligodendrocyte death results in immune-mediated CNS demyelination. Nat. Neurosci..

[102] Caprariello A.V., Mangla S., Miller R.H., Selkirk S.M. (2012). Apoptosis of oligodendrocytes in the central nervous system results in rapid focal demyelination. Ann. Neurol..

[103] Prineas J.W., Parratt J.D. (2012). Oligodendrocytes and the early multiple sclerosis lesion. Ann. Neurol..

[104] Caprariello A.V., Batt C.E., Zippe I., Romito-DiGiacomo R.R., Karl M., Miller R.H. (2015). Apoptosis of Oligodendrocytes during Early Development Delays Myelination and Impairs Subsequent Responses to Demyelination. J. Neurosci..

[105] Hövelmeyer N., Hao Z., Kranidioti K., Kassiotis G., Buch T., Frommer F., von Hoch L., Kramer D., Minichiello L., Kollias G. (2005). Apoptosis of oligodendrocytes via Fas and TNF-R1 is a key event in the induction of experimental autoimmune encephalomyelitis. J. Immunol..

[106] Jurewicz A., Matysiak M., Tybor K., Kilianek L., Raine C.S., Selmaj K. (2005). Tumour necrosis factor-induced death of adult human oligodendrocytes is mediated by apoptosis inducing factor. Brain.

[107] Seil F.J. (2018). Myelin Antigens and Antimyelin Antibodies. Antibodies.

[108] Eliseeva D.D., Zakharova M.N. (2023). Myelin Oligodendrocyte Glycoprotein as an Autoantigen in Inflammatory Demyelinating Diseases of the Central Nervous System. Biochemistry.

[109] Hedegaard C.J., Chen N., Sellebjerg F., Sørensen P.S., Leslie R.G., Bendtzen K., Nielsen C.H. (2009). Autoantibodies to myelin basic protein (MBP) in healthy individuals and in patients with multiple sclerosis: A role in regulating cytokine responses to MBP. Immunology.

[110] Bittner S., Meuth S.G. (2013). Targeting ion channels for the treatment of autoimmune neuroinflammation. Ther. Adv. Neurol. Disord..

[111] Nicaise C., Marneffe C., Bouchat J., Gilloteaux J. (2019). Osmotic Demyelination: From an Oligodendrocyte to an Astrocyte Perspective. Int. J. Mol. Sci..

[112] Ofengeim D., Ito Y., Najafov A., Zhang Y., Shan B., DeWitt J.P., Ye J., Zhang X., Chang A., Vakifahmetoglu-Norberg H. (2015). Activation of necroptosis in multiple sclerosis. Cell Rep..

[113] Yuan J., Amin P., Ofengeim D. (2019). Necroptosis and RIPK1-mediated neuroinflammation in CNS diseases. Nat. Rev. Neurosci..

[114] Zhou Y., Xiang Y., Liu S., Li C., Dong J., Kong X., Ji X., Cheng X., Zhang L. (2024). RIPK3 signaling and its role in regulated cell death and diseases. Cell Death Discov..

[115] Xiao M., Gao G., Mu J., Sun Q., Zhao Y., Fan X. (2025). MLKL Modulates Necroptosis and Neuroinflammation in a Mouse Model of MS. Inflammation.

[116] Faergeman S.L., Evans H., Attfield K.E., Desel C., Kuttikkatte S.B., Sommerlund M., Jensen L.T., Frokiaer J., Friese M.A., Matthews P.M. (2020). A novel neurodegenerative spectrum disorder in patients with MLKL deficiency. Cell Death Dis..

[117] Huntemer-Silveira A., Patil N., Brickner M.A., Parr A.M. (2021). Strategies for Oligodendrocyte and Myelin Repair in Traumatic CNS Injury. Front. Cell Neurosci..

[118] Duncan I.D., Radcliff A.B., Heidari M., Kidd G., August B.K., Wierenga L.A. (2018). The adult oligodendrocyte can participate in remyelination. Proc. Natl. Acad. Sci. USA.

[119] Mezydlo A., Treiber N., Ullrich Gavilanes E.M., Eichenseer K., Ancău M., Wens A., Ares Carral C., Schifferer M., Snaidero N., Misgeld T. (2023). Remyelination by surviving oligodendrocytes is inefficient in the inflamed mammalian cortex. Neuron.

[120] Gharagozloo M., Bannon R., Calabresi P.A. (2022). Breaking the barriers to remyelination in multiple sclerosis. Curr. Opin. Pharmacol..

[121] Tiane A., Schepers M., Rombaut B., Hupperts R., Prickaerts J., Hellings N., van den Hove D., Vanmierlo T. (2019). From OPC to Oligodendrocyte: An Epigenetic Journey. Cells.

[122] Emery B., Lu Q.R. (2015). Transcriptional and Epigenetic Regulation of Oligodendrocyte Development and Myelination in the Central Nervous System. Cold Spring Harb. Perspect. Biol..

[123] Hernandez M., Casaccia P. (2015). Interplay between transcriptional control and chromatin regulation in the oligodendrocyte lineage. GLIA.

[124] Ye F., Chen Y., Hoang T., Montgomery R.L., Zhao X.H., Bu H., Hu T., Taketo M.M., van Es J.H., Clevers H. (2009). HDAC1 and HDAC2 regulate oligodendrocyte differentiation by disrupting the beta-catenin-TCF interaction. Nat. Neurosci..

[125] Castro K., Casaccia P. (2018). Epigenetic modifications in brain and immune cells of multiple sclerosis patients. Mult. Scler..

[126] John G.R., Shankar S.L., Shafit-Zagardo B., Massimi A., Lee S.C., Raine C.S., Brosnan C.F. (2002). Multiple sclerosis: Re-expression of a developmental pathway that restricts oligodendrocyte maturation. Nat. Med..

[127] Allan K.C., Miller T.E., Morton A.R., Scavuzzo M.A., Elitt M.S., Clayton B.L.L., Hu L.R., Vrabic J.K., Olsen H.E., Factor D.C. (2022). Cellular Maturation of Oligodendrocytes is Governed by Transient Gene Melting. bioRxiv.

[128] Dai Z.M., Sun S., Wang C., Huang H., Hu X., Zhang Z., Lu Q.R., Qiu M. (2014). Stage-specific regulation of oligodendrocyte development by Wnt/β-catenin signaling. J. Neurosci..

[129] Hammond E., Lang J., Maeda Y., Pleasure D., Angus-Hill M., Xu J., Horiuchi M., Deng W., Guo F. (2015). The Wnt effector transcription factor 7-like 2 positively regulates oligodendrocyte differentiation in a manner independent of Wnt/β-catenin signaling. J. Neurosci..

[130] Yu Y., Casaccia P., Lu Q.R. (2010). Shaping the oligodendrocyte identity by epigenetic control. Epigenetics.

[131] Juryńczyk M., Selmaj K. (2010). Notch: A new player in MS mechanisms. J. Neuroimmunol..

[132] Wu M., Hernandez M., Shen S., Sabo J.K., Kelkar D., Wang J., O’Leary R., Phillips G.R., Cate H.S., Casaccia P. (2012). Differential modulation of the oligodendrocyte transcriptome by sonic hedgehog and bone morphogenetic protein 4 via opposing effects on histone acetylation. J. Neurosci..

[133] Haase S., Linker R.A. (2021). Inflammation in multiple sclerosis. Ther. Adv. Neurol. Disord..

[134] Itoh T., Horiuchi M., Itoh A. (2009). Interferon-triggered transcriptional cascades in the oligodendroglial lineage: A comparison of induction of MHC class II antigen between oligodendroglial progenitor cells and mature oligodendrocytes. J. Neuroimmunol..

[135] Brambilla R., Ashbaugh J.J., Magliozzi R., Dellarole A., Karmally S., Szymkowski D.E., Bethea J.R. (2011). Inhibition of soluble tumour necrosis factor is therapeutic in experimental autoimmune encephalomyelitis and promotes axon preservation and remyelination. Brain.

[136] Raasch J., Zeller N., van Loo G., Merkler D., Mildner A., Erny D., Knobeloch K.P., Bethea J.R., Waisman A., Knust M. (2011). IkappaB kinase 2 determines oligodendrocyte loss by non-cell-autonomous activation of NF-kappaB in the central nervous system. Brain.

[137] Horellou P., Wang M., Keo V., Chrétien P., Serguera C., Waters P., Deiva K. (2015). Increased interleukin-6 correlates with myelin oligodendrocyte glycoprotein antibodies in pediatric monophasic demyelinating diseases and multiple sclerosis. J. Neuroimmunol..

[138] Lang B.T., Cregg J.M., DePaul M.A., Tran A.P., Xu K., Dyck S.M., Madalena K.M., Brown B.P., Weng Y.L., Li S. (2015). Modulation of the proteoglycan receptor PTPσ promotes recovery after spinal cord injury. Nature.

[139] Ghorbani S., Yong V.W. (2021). The extracellular matrix as modifier of neuroinflammation and remyelination in multiple sclerosis. Brain.

[140] Wei S.S., Chen L., Yang F.Y., Wang S.Q., Wang P. (2023). The role of fibronectin in multiple sclerosis and the effect of drug delivery across the blood-brain barrier. Neural Regen. Res..

[141] Back S.A., Tuohy T.M., Chen H., Wallingford N., Craig A., Struve J., Luo N.L., Banine F., Liu Y., Chang A. (2005). Hyaluronan accumulates in demyelinated lesions and inhibits oligodendrocyte progenitor maturation. Nat. Med..

[142] Bauch J., Faissner A. (2022). The Extracellular Matrix Proteins Tenascin-C and Tenascin-R Retard Oligodendrocyte Precursor Maturation and Myelin Regeneration in a Cuprizone-Induced Long-Term Demyelination Animal Model. Cells.

[143] López-Muguruza E., Matute C. (2023). Alterations of Oligodendrocyte and Myelin Energy Metabolism in Multiple Sclerosis. Int. J. Mol. Sci..

[144] Gao R., Song S.J., Tian M.Y., Wang L.B., Zhang Y., Li X. (2024). Myelin debris phagocytosis in demyelinating disease. GLIA.

[145] Rosko L., Smith V.N., Yamazaki R., Huang J.K. (2019). Oligodendrocyte Bioenergetics in Health and Disease. Neuroscientist.

[146] Narine M., Colognato H. (2022). Current Insights Into Oligodendrocyte Metabolism and Its Power to Sculpt the Myelin Landscape. Front. Cell Neurosci..

[147] Liu H., Wang S., Wang J., Guo X., Song Y., Fu K., Gao Z., Liu D., He W., Yang L.L. (2025). Energy metabolism in health and diseases. Signal Transduct. Target. Ther..

[148] Bonora M., De Marchi E., Patergnani S., Suski J.M., Celsi F., Bononi A., Giorgi C., Marchi S., Rimessi A., Duszyński J. (2014). Tumor necrosis factor-α impairs oligodendroglial differentiation through a mitochondria-dependent process. Cell Death Differ..

[149] Ziabreva I., Campbell G., Rist J., Zambonin J., Rorbach J., Wydro M.M., Lassmann H., Franklin R.J., Mahad D. (2010). Injury and differentiation following inhibition of mitochondrial respiratory chain complex IV in rat oligodendrocytes. GLIA.

[150] Duncan G.J., Simkins T.J., Emery B. (2021). Neuron-Oligodendrocyte Interactions in the Structure and Integrity of Axons. Front. Cell Dev. Biol..

[151] Spaas J., van Veggel L., Schepers M., Tiane A., van Horssen J., Wilson D.M., Moya P.R., Piccart E., Hellings N., Eijnde B.O. (2021). Oxidative stress and impaired oligodendrocyte precursor cell differentiation in neurological disorders. Cell. Mol. Life Sci..

[152] Baud O., Haynes R.F., Wang H., Folkerth R.D., Li J., Volpe J.J., Rosenberg P.A. (2004). Developmental up-regulation of MnSOD in rat oligodendrocytes confers protection against oxidative injury. Eur. J. Neurosci..

[153] Lipinski B. (2011). Hydroxyl radical and its scavengers in health and disease. Oxid. Med. Cell Longev..

[154] Nissanka N., Moraes C.T. (2018). Mitochondrial DNA damage and reactive oxygen species in neurodegenerative disease. FEBS Lett..

[155] Xu X., Pang Y., Fan X. (2025). Mitochondria in oxidative stress, inflammation and aging: From mechanisms to therapeutic advances. Signal Transduct. Target. Ther..

[156] Haider L., Fischer M.T., Frischer J.M., Bauer J., Höftberger R., Botond G., Esterbauer H., Binder C.J., Witztum J.L., Lassmann H. (2011). Oxidative damage in multiple sclerosis lesions. Brain.

[157] Campbell G., Mahad D.J. (2018). Mitochondrial dysfunction and axon degeneration in progressive multiple sclerosis. FEBS Lett..

[158] van den Berg R., Hoogenraad C.C., Hintzen R.Q. (2017). Axonal transport deficits in multiple sclerosis: Spiraling into the abyss. Acta Neuropathol..

[159] Zhai D., Yan S., Samsom J., Wang L., Su P., Jiang A., Zhang H., Jia Z., Wallach I., Heifets A. (2023). Small-molecule targeting AMPA-mediated excitotoxicity has therapeutic effects in mouse models for multiple sclerosis. Sci. Adv..

[160] Hill K.E., Zollinger L.V., Watt H.E., Carlson N.G., Rose J.W. (2004). Inducible nitric oxide synthase in chronic active multiple sclerosis plaques: Distribution, cellular expression and association with myelin damage. J. Neuroimmunol..

[161] Li S., Lin W., Tchantchou F., Lai R., Wen J., Zhang Y. (2011). Protein kinase C mediates peroxynitrite toxicity to oligodendrocytes. Mol. Cell. Neurosci..

[162] Kukanja P., Langseth C.M., Rubio Rodríguez-Kirby L.A., Agirre E., Zheng C., Raman A., Yokota C., Avenel C., Tiklová K., Guerreiro-Cacais A.O. (2024). Cellular architecture of evolving neuroinflammatory lesions and multiple sclerosis pathology. Cell.

[163] Lerma-Martin C., Badia-I.-Mompel P., Ramirez Flores R.O., Sekol P., Schäfer P.S.L., Riedl C.J., Hofmann A., Thäwel T., Wünnemann F., Ibarra-Arellano M.A. (2024). Cell type mapping reveals tissue niches and interactions in subcortical multiple sclerosis lesions. Nat. Neurosci..

[164] Hendrickx D.A.E., van Scheppingen J., van der Poel M., Bossers K., Schuurman K.G., van Eden C.G., Hol E.M., Hamann J., Huitinga I. (2017). Gene Expression Profiling of Multiple Sclerosis Pathology Identifies Early Patterns of Demyelination Surrounding Chronic Active Lesions. Front. Immunol..

[165] Riekkinen P.J., Palo J., Arstila A.U., Savolainen H.J., Rinne U.K., Kivalo E.K., Frey H. (1971). Protein composition of multiple sclerosis myelin. Arch. Neurol..

[166] Raasakka A., Kursula P. (2020). Flexible Players within the Sheaths: The Intrinsically Disordered Proteins of Myelin in Health and Disease. Cells.

[167] Chen Y., Kunjamma R.B., Weiner M., Chan J.R., Popko B. (2021). Prolonging the integrated stress response enhances CNS remyelination in an inflammatory environment. eLife.

[168] Maghbooli Z., Sayahpour F.A., Varzandi T., Masoumi M.R., Sahraian M.A. (2025). The interplay between endoplasmic reticulum stress and inflammation in multiple sclerosis. Sci. Rep..

[169] Stone S., Lin W. (2015). The unfolded protein response in multiple sclerosis. Front. Neurosci..

[170] Lin W. (2015). Impaired eIF2B activity in ligodendrocytes contributes to VWMD pathogenesis. Neural Regen. Res..

[171] Junjappa R.P., Patil P., Bhattarai K.R., Kim H.R., Chae H.J. (2018). IRE1α Implications in Endoplasmic Reticulum Stress-Mediated Development and Pathogenesis of Autoimmune Diseases. Front. Immunol..

[172] Stone S., Wu S., Jamison S., Durose W., Pallais J.P., Lin W. (2018). Activating transcription factor 6α deficiency exacerbates oligodendrocyte death and myelin damage in immune-mediated demyelinating diseases. GLIA.

[173] Mháille A.N., McQuaid S., Windebank A., Cunnea P., McMahon J., Samali A., FitzGerald U. (2008). Increased expression of endoplasmic reticulum stress-related signaling pathway molecules in multiple sclerosis lesions. J. Neuropathol. Exp. Neurol..

[174] Yoshida H., Matsui T., Yamamoto A., Okada T., Mori K. (2001). XBP1 mRNA is induced by ATF6 and spliced by IRE1 in response to ER stress to produce a highly active transcription factor. Cell.

[175] Lei Y., Yu H., Ding S., Liu H., Liu C., Fu R. (2024). Molecular mechanism of ATF6 in unfolded protein response and its role in disease. Heliyon.

[176] Lin W., Stone S. (2020). Unfolded protein response in myelin disorders. Neural Regen. Res..

[177] Sharma G., Gopinath S., Lakshmi Narasimhan R. (2022). Exploring the Molecular Aspects of Glycosylation in MOG Antibody Disease (MOGAD). Curr. Protein Pept. Sci..

[178] Feigenson K., Reid M., See J., Crenshaw E.B., Grinspan J.B. (2009). Wnt signaling is sufficient to perturb oligodendrocyte maturation. Mol. Cell. Neurosci..

[179] Fancy S.P., Baranzini S.E., Zhao C., Yuk D.I., Irvine K.A., Kaing S., Sanai N., Franklin R.J., Rowitch D.H. (2009). Dysregulation of the Wnt pathway inhibits timely myelination and remyelination in the mammalian CNS. Genes. Dev..

[180] Shimizu T., Kagawa T., Wada T., Muroyama Y., Takada S., Ikenaka K. (2005). Wnt signaling controls the timing of oligodendrocyte development in the spinal cord. Dev. Biol..

[181] Huang S.M., Mishina Y.M., Liu S., Cheung A., Stegmeier F., Michaud G.A., Charlat O., Wiellette E., Zhang Y., Wiessner S. (2009). Tankyrase inhibition stabilizes axin and antagonizes Wnt signalling. Nature.

[182] Fancy S.P., Harrington E.P., Yuen T.J., Silbereis J.C., Zhao C., Baranzini S.E., Bruce C.C., Otero J.J., Huang E.J., Nusse R. (2011). Axin2 as regulatory and therapeutic target in newborn brain injury and remyelination. Nat. Neurosci..

[183] Chen J., Li J., Miao Z., Xu X., Liu C.F. (2014). XAV939, a small molecular inhibitor, provides neuroprotective effects on oligodentrocytes. J. Neurosci. Res..

[184] Shih Y., Ly P.T.T., Wang J., Pallen C.J. (2017). Glial and Neuronal Protein Tyrosine Phosphatase Alpha (PTPα) Regulate Oligodendrocyte Differentiation and Myelination. J. Mol. Neurosci..

[185] Li Y., Liu L., Ding X., Liu Y., Yang Q., Ren B. (2020). Interleukin-1β attenuates the proliferation and differentiation of oligodendrocyte precursor cells through regulation of the microRNA-202-3p/β-catenin/Gli1 axis. Int. J. Mol. Med..

[186] Zhang N., Zhang S., Liu X., Zuo Y.Y., Cui Y.G., Wang F., Zhang J.H., Chang Y.Z., Yu P. (2025). Oligodendrocyte-specific knockout of FPN1 affects CNS myelination defects and depression-like behavior in mice. Free Radic. Biol. Med..

[187] Russo M., Zahaf A., Kassoussi A., Sharif A., Faure H., Traiffort E., Ruat M. (2024). Sonic Hedgehog Is an Early Oligodendrocyte Marker During Remyelination. Cells.

[188] Cohen M., Kicheva A., Ribeiro A., Blassberg R., Page K.M., Barnes C.P., Briscoe J. (2015). Ptch1 and Gli regulate Shh signalling dynamics via multiple mechanisms. Nat. Commun..

[189] Prajapati A., Mehan S., Khan Z., Chhabra S., Das Gupta G. (2024). Purmorphamine, a Smo-Shh/Gli Activator, Promotes Sonic Hedgehog-Mediated Neurogenesis and Restores Behavioural and Neurochemical Deficits in Experimental Model of Multiple Sclerosis. Neurochem. Res..

[190] Namchaiw P., Wen H., Mayrhofer F., Chechneva O., Biswas S., Deng W. (2019). Temporal and partial inhibition of GLI1 in neural stem cells (NSCs) results in the early maturation of NSC derived oligodendrocytes in vitro. Stem Cell Res. Ther..

[191] Porcu G., Serone E., De Nardis V., Di Giandomenico D., Lucisano G., Scardapane M., Poma A., Ragnini-Wilson A. (2015). Clobetasol and Halcinonide Act as Smoothened Agonists to Promote Myelin Gene Expression and RxRγ Receptor Activation. PLoS ONE..

[192] Yao X., Su T., Verkman A.S. (2016). Clobetasol promotes remyelination in a mouse model of neuromyelitis optica. Acta Neuropathol. Commun..

[193] Wang S., Sdrulla A.D., diSibio G., Bush G., Nofziger D., Hicks C., Weinmaster G., Barres B.A. (1998). Notch receptor activation inhibits oligodendrocyte differentiation. Neuron.

[194] Zhang Y., Argaw A.T., Gurfein B.T., Zameer A., Snyder B.J., Ge C., Lu Q.R., Rowitch D.H., Raine C.S., Brosnan C.F. (2009). Notch1 signaling plays a role in regulating precursor differentiation during CNS remyelination. Proc. Natl. Acad. Sci. USA.

[195] Hammond T.R., Gadea A., Dupree J., Kerninon C., Nait-Oumesmar B., Aguirre A., Gallo V. (2014). Astrocyte-derived endothelin-1 inhibits remyelination through notch activation. Neuron.

[196] Brosnan C.F., John G.R. (2009). Revisiting Notch in remyelination of multiple sclerosis lesions. J. Clin. Investig..

[197] Wang C., Zhang C.J., Martin B.N., Bulek K., Kang Z., Zhao J., Bian G., Carman J.A., Gao J., Dongre A. (2017). IL-17 induced NOTCH1 activation in oligodendrocyte progenitor cells enhances proliferation and inflammatory gene expression. Nat. Commun..

[198] Jurynczyk M., Jurewicz A., Bielecki B., Raine C.S., Selmaj K. (2005). Inhibition of Notch signaling enhances tissue repair in an animal model of multiple sclerosis. J. Neuroimmunol..

[199] Fletcher J.L., Wood R.J., Nguyen J., Norman E.M.L., Jun C.M.K., Prawdiuk A.R., Biemond M., Nguyen H.T.H., Northfield S.E., Hughes R.A. (2018). Targeting TrkB with a Brain-Derived Neurotrophic Factor Mimetic Promotes Myelin Repair in the Brain. J. Neurosci..

[200] Xiao J. (2023). Thirty years of BDNF study in central myelination: From biology to therapy. J. Neurochem..

[201] Makar T.K., Nimmagadda V.K., Singh I.S., Lam K., Mubariz F., Judge S.I., Trisler D., Bever C.T. (2016). TrkB agonist, 7,8-dihydroxyflavone, reduces the clinical and pathological severity of a murine model of multiple sclerosis. J. Neuroimmunol..

[202] Al-Samerria S., Radovick S. (2021). The Role of Insulin-like Growth Factor-1 (IGF-1) in the Control of Neuroendocrine Regulation of Growth. Cells.

[203] Zeger M., Popken G., Zhang J., Xuan S., Lu Q.R., Schwab M.H., Nave K.A., Rowitch D., D’Ercole A.J., Ye P. (2007). Insulin-like growth factor type 1 receptor signaling in the cells of oligodendrocyte lineage is required for normal in vivo oligodendrocyte development and myelination. GLIA.

[204] Bibollet-Bahena O., Almazan G. (2009). IGF-1-stimulated protein synthesis in oligodendrocyte progenitors requires PI3K/mTOR/Akt and MEK/ERK pathways. J. Neurochem..

[205] Yao D.L., Liu X., Hudson L.D., Webster H.D. (1995). Insulin-like growth factor I treatment reduces demyelination and up-regulates gene expression of myelin-related proteins in experimental autoimmune encephalomyelitis. Proc. Natl. Acad. Sci. USA.

[206] Ye P., D’Ercole A.J. (1999). Insulin-like growth factor I protects oligodendrocytes from tumor necrosis factor-alpha-induced injury. Endocrinology.

[207] Hlavica M., Delparente A., Good A., Good N., Plattner P.S., Seyedsadr M.S., Schwab M.E., Figlewicz D.P., Ineichen B.V. (2017). Intrathecal insulin-like growth factor 1 but not insulin enhances myelin repair in young and aged rats. Neurosci. Lett..

[208] Brinkmann B.G., Agarwal A., Sereda M.W., Garratt A.N., Müller T., Wende H., Stassart R.M., Nawaz S., Humml C., Velanac V. (2008). Neuregulin-1/ErbB signaling serves distinct functions in myelination of the peripheral and central nervous system. Neuron.

[209] Galvez-Contreras A.Y., Quiñones-Hinojosa A., Gonzalez-Perez O. (2013). The role of EGFR and ErbB family related proteins in the oligodendrocyte specification in germinal niches of the adult mammalian brain. Front. Cell Neurosci..

[210] Mei L., Nave K.A. (2014). Neuregulin-ERBB signaling in the nervous system and neuropsychiatric diseases. Neuron.

[211] Xu C., Lv L., Zheng G., Li B., Gao L., Sun Y. (2012). Neuregulin1β1 protects oligodendrocyte progenitor cells from oxygen glucose deprivation injury induced apoptosis via ErbB4-dependent activation of PI3-kinase/Akt. Brain Res..

[212] Kataria H., Alizadeh A., Shahriary G.M., Saboktakin Rizi S., Henrie R., Santhosh K.T., Thliveris J.A., Karimi-Abdolrezaee S. (2018). Neuregulin-1 promotes remyelination and fosters a pro-regenerative inflammatory response in focal demyelinating lesions of the spinal cord. GLIA.

[213] Gregath A., Lu Q.R. (2018). Epigenetic modifications-insight into oligodendrocyte lineage progression, regeneration, and disease. FEBS Lett..

[214] Shukla S., Tekwani B.L. (2020). Histone Deacetylases Inhibitors in Neurodegenerative Diseases, Neuroprotection and Neuronal Differentiation. Front. Pharmacol..

[215] Pazhoohan S., Satarian L., Asghari A.A., Salimi M., Kiani S., Mani A.R., Javan M. (2014). Valproic Acid attenuates disease symptoms and increases endogenous myelin repair by recruiting neural stem cells and oligodendrocyte progenitors in experimental autoimmune encephalomyelitis. Neurodegener. Dis..

[216] Castelo-Branco G., Stridh P., Guerreiro-Cacais A.O., Adzemovic M.Z., Falcão A.M., Marta M., Berglund R., Gillett A., Hamza K.H., Lassmann H. (2014). Acute treatment with valproic acid and l-thyroxine ameliorates clinical signs of experimental autoimmune encephalomyelitis and prevents brain pathology in DA rats. Neurobiol. Dis..

[217] Rossi M., Petralla S., Protti M., Baiula M., Kobrlova T., Soukup O., Spampinato S.M., Mercolini L., Monti B., Bolognesi M.L. (2020). α-Linolenic Acid-Valproic Acid Conjugates: Toward Single-Molecule Polypharmacology for Multiple Sclerosis. ACS Med. Chem. Lett..

[218] Rivera A.D., Pieropan F., Williams G., Calzolari F., Butt A.M., Azim K. (2022). Drug connectivity mapping and functional analysis reveal therapeutic small molecules that differentially modulate myelination. Biomed. Pharmacother..

[219] Motavaf M., Sadeghizadeh M., Babashah S., Zare L., Javan M. (2020). Protective Effects of a Nano-Formulation of Curcumin against Cuprizone-Induced Demyelination in the Mouse Corpus Callosum. Iran. J. Pharm. Res..

[220] Bernardo A., Plumitallo C., De Nuccio C., Visentin S., Minghetti L. (2021). Curcumin promotes oligodendrocyte differentiation and their protection against TNF-α through the activation of the nuclear receptor PPAR-γ. Sci. Rep..

[221] Ren X., Yang Y., Wang M., Yuan Q., Suo N., Xie X. (2024). Vitamin C and MEK Inhibitor PD0325901 Synergistically Promote Oligodendrocytes Generation by Promoting DNA Demethylation. Molecules.

[222] Ngo C., Kothary R. (2022). MicroRNAs in oligodendrocyte development and remyelination. J. Neurochem..

[223] Afrang N., Tavakoli R., Tasharrofi N., Alian A., Naderi Sohi A., Kabiri M., Fathi-Roudsari M., Soufizomorrod M., Rajaei F., Soleimani M. (2019). A critical role for miR-184 in the fate determination of oligodendrocytes. Stem Cell Res. Ther..

[224] Perdaens O., Bottemanne P., van Pesch V. (2024). MicroRNAs dysregulated in multiple sclerosis affect the differentiation of CG-4 cells, an oligodendrocyte progenitor cell line. Front. Cell Neurosci..

[225] Xie C., Liu Y.Q., Guan Y.T., Zhang G.X. (2016). Induced Stem Cells as a Novel Multiple Sclerosis Therapy. Curr. Stem Cell Res. Ther..

[226] Fortune A.J., Fletcher J.L., Blackburn N.B., Young K.M. (2022). Using MS induced pluripotent stem cells to investigate MS aetiology. Mult. Scler. Relat. Disord..

[227] Morales Pantoja I.E., Smith M.D., Rajbhandari L., Cheng L., Gao Y., Mahairaki V., Venkatesan A., Calabresi P.A., Fitzgerald K.C., Whartenby K.A. (2020). iPSCs from people with MS can differentiate into oligodendrocytes in a homeostatic but not an inflammatory milieu. PLoS ONE.

[228] Martinez-Curiel R., Jansson L., Tsupykov O., Avaliani N., Aretio-Medina C., Hidalgo I., Monni E., Bengzon J., Skibo G., Lindvall O. (2023). Oligodendrocytes in human induced pluripotent stem cell-derived cortical grafts remyelinate adult rat and human cortical neurons. Stem Cell Rep..

[229] Sharp J., Keirstead H.S. (2007). Therapeutic applications of oligodendrocyte precursors derived from human embryonic stem cells. Curr. Opin. Biotechnol..

[230] Alsanie W.F., Niclis J.C., Petratos S. (2013). Human embryonic stem cell-derived oligodendrocytes: Protocols and perspectives. Stem Cells Dev..

[231] Douvaras P., Wang J., Zimmer M., Hanchuk S., O’Bara M.A., Sadiq S., Sim F.J., Goldman J., Fossati V. (2014). Efficient generation of myelinating oligodendrocytes from primary progressive multiple sclerosis patients by induced pluripotent stem cells. Stem Cell Rep..

[232] Yamashita T., Miyamoto Y., Bando Y., Ono T., Kobayashi S., Doi A., Araki T., Kato Y., Shirakawa T., Suzuki Y. (2017). Differentiation of oligodendrocyte progenitor cells from dissociated monolayer and feeder-free cultured pluripotent stem cells. PLoS ONE.

[233] Wang Q., Huang T., Zheng Z., Su Y., Wu Z., Zeng C., Yu G., Liu Y., Wang X., Li H. (2025). Oligodendroglial precursor cells modulate immune response and early demyelination in a murine model of multiple sclerosis. Sci. Transl. Med..

[234] Meco E., Lampe K.J. (2018). Microscale Architecture in Biomaterial Scaffolds for Spatial Control of Neural Cell Behavior. Front. Mater..

[235] Mazur R.A., Lampe K.J. (2025). Guiding Oligodendrocyte Progenitor Cell Maturation Using Electrospun Fiber Cues in a 3D Hyaluronic Acid Hydrogel Culture System. ACS Biomater. Sci. Eng..

[236] Luo T., Tan B., Zhu L., Wang Y., Liao J. (2022). A Review on the Design of Hydrogels With Different Stiffness and Their Effects on Tissue Repair. Front. Bioeng. Biotechnol..

[237] Wang Y., Tan H., Hui X. (2018). Biomaterial Scaffolds in Regenerative Therapy of the Central Nervous System. Biomed. Res. Int..

[238] Lager A.M., Corradin O.G., Cregg J.M., Elitt M.S., Shick H.E., Clayton B.L.L., Allan K.C., Olsen H.E., Madhavan M., Tesar P.J. (2018). Rapid functional genetics of the oligodendrocyte lineage using pluripotent stem cells. Nat. Commun..

[239] Wagstaff L.J., Bestard-Cuche N., Kaczmarek M., Fidanza A., McNeil L., Franklin R.J.M., Williams A.C. (2024). CRISPR-edited human ES-derived oligodendrocyte progenitor cells improve remyelination in rodents. Nat. Commun..

[240] Romero J.C., Berlinicke C., Chow S., Duan Y., Wang Y., Chamling X., Smirnova L. (2023). Oligodendrogenesis and myelination tracing in a CRISPR/Cas9-engineered brain microphysiological system. Front. Cell Neurosci..

[241] Feng L., Chao J., Ye P., Luong Q., Sun G., Liu W., Cui Q., Flores S., Jackson N., Shayento A.N.H. (2023). Developing Hypoimmunogenic Human iPSC-Derived Oligodendrocyte Progenitor Cells as an Off-The-Shelf Cell Therapy for Myelin Disorders. Adv. Sci..

[242] Blaszczyk G.J., Mohammadnia A., Piscopo V.E.C., Sirois J., Cui Q.L., Yaqubi M., Durcan T.M., Schneider R., Antel J.P. (2025). Pro-Inflammatory Molecules Implicated in Multiple Sclerosis Divert the Development of Human Oligodendrocyte Lineage Cells. Neurol. Neuroimmunol. Neuroinflamm..

[243] Bernardo A., Bianchi D., Magnaghi V., Minghetti L. (2009). Peroxisome proliferator-activated receptor-gamma agonists promote differentiation and antioxidant defenses of oligodendrocyte progenitor cells. J. Neuropathol. Exp. Neurol..

[244] Maier K., Merkler D., Gerber J., Taheri N., Kuhnert A.V., Williams S.K., Neusch C., Bähr M., Diem R. (2007). Multiple neuroprotective mechanisms of minocycline in autoimmune CNS inflammation. Neurobiol. Dis..

[245] Marzan D.E., Brügger-Verdon V., West B.L., Liddelow S., Samanta J., Salzer J.L. (2021). Activated microglia drive demyelination via CSF1R signaling. GLIA.

[246] Tahmasebi F., Barati S., Kashani I.R. (2021). Effect of CSF1R inhibitor on glial cells population and remyelination in the cuprizone model. Neuropeptides.

[247] Shirvanchi K., Gurski F., Rajendran V., Rajendran R., Megalofonou F.F., Stadelmann-Nessler C., Karnati S., Berghoff M. (2024). The Multi-kinase Inhibitor AZD4547 Reduces Inflammation and Neurodegeneration, and Enhances Remyelination in a Mouse Model of Multiple Sclerosis (P7-6.008). Neurology.

[248] Cui Y., Yu H., Bu Z., Wen L., Yan L., Feng J. (2022). Focus on the Role of the NLRP3 Inflammasome in Multiple Sclerosis: Pathogenesis, Diagnosis, and Therapeutics. Front. Mol. Neurosci..

[249] Zhang W.G., Zheng X.R., Yao Y., Sun W.J., Shao B.Z. (2025). The role of NLRP3 inflammasome in multiple sclerosis: Pathogenesis and pharmacological application. Front. Immunol..

[250] Hou B., Yin J., Liu S., Guo J., Zhang B., Zhang Z., Yang L., Tan X., Long Y., Feng S. (2024). Inhibiting the NLRP3 Inflammasome with MCC950 Alleviates Neurological Impairment in the Brain of EAE Mice. Mol. Neurobiol..

[251] Rashidbenam Z., Ozturk E., Pagnin M., Theotokis P., Grigoriadis N., Petratos S. (2023). How does Nogo receptor influence demyelination and remyelination in the context of multiple sclerosis?. Front. Cell Neurosci..

[252] Kalafatakis I., Papagianni F., Theodorakis K., Karagogeos D. (2023). Nogo-A and LINGO-1: Two Important Targets for Remyelination and Regeneration. Int. J. Mol. Sci..

[253] Pernet V., Joly S., Spiegel S., Meli I., Idriss S., Maigler F., Mdzomba J.B., Roenneke A.K., Franceschini A., Silvestri L. (2023). Nogo-A antibody delivery through the olfactory mucosa mitigates experimental autoimmune encephalomyelitis in the mouse CNS. Cell Death Discov..

[254] Sun J.J., Ren Q.G., Xu L., Zhang Z.J. (2015). LINGO-1 antibody ameliorates myelin impairment and spatial memory deficits in experimental autoimmune encephalomyelitis mice. Sci. Rep..

[255] Moradbeygi K., Parviz M., Rezaeizadeh H., Zargaran A., Sahraian M.A., Mehrabadi S., Nikbakhtzadeh M., Zahedi E. (2021). Anti-LINGO-1 improved remyelination and neurobehavioral deficit in cuprizone-induced demyelination. Iran. J. Basic. Med. Sci..

[256] Aktas O., Ziemssen F., Ziemssen T., Klistorner A., Butzkueven H., Izquierdo G., Leocani L., Balcer L.J., Galetta S.L., Castrillo-Viguera C. (2025). RENEWED: A follow-up study of the opicinumab phase 2 RENEW study in participants with acute optic neuritis. Mult. Scler. Relat. Disord..

[257] Amadio S., Conte F., Esposito G., Fiscon G., Paci P., Volonté C. (2022). Repurposing Histaminergic Drugs in Multiple Sclerosis. Int. J. Mol. Sci..

[258] Motawi T.K., El-Maraghy S.A., Kamel A.S., Said S.E., Kortam M.A. (2023). Modulation of p38 MAPK and Nrf2/HO-1/NLRP3 inflammasome signaling and pyroptosis outline the anti-neuroinflammatory and remyelinating characters of Clemastine in EAE rat model. Biochem. Pharmacol..

[259] Ibrahim S.M., Kamel A.S., Ahmed K.A., Mohammed R.A., Essam R.M. (2024). The preferential effect of Clemastine on F3/Contactin-1/Notch-1 compared to Jagged-1/Notch-1 justifies its remyelinating effect in an experimental model of multiple sclerosis in rats. Int. Immunopharmacol..

[260] Cui Q.L., Fogle E., Almazan G. (2006). Muscarinic acetylcholine receptors mediate oligodendrocyte progenitor survival through Src-like tyrosine kinases and PI3K/Akt pathways. Neurochem. Int..

[261] Chen K., Park E., Abd-Elrahman K.S. (2025). Enhancing remyelination in multiple sclerosis via M1 muscarinic acetylcholine receptor. Mol. Pharmacol..

[262] Poon M.M., Lorrain K.I., Stebbins K.J., Edu G.C., Broadhead A.R., Lorenzana A.J., Roppe J.R., Baccei J.M., Baccei C.S., Chen A.C. (2024). Targeting the muscarinic M1 receptor with a selective, brain-penetrant antagonist to promote remyelination in multiple sclerosis. Proc. Natl. Acad. Sci. USA.

[263] Poon M.M., Lorrain K.I., Stebbins K.J., Edu G.C., Broadhead A.R., Lorenzana A.O., Paulson B.E., Baccei C.S., Roppe J.R., Schrader T.O. (2024). Discovery of a brain penetrant small molecule antagonist targeting LPA1 receptors to reduce neuroinflammation and promote remyelination in multiple sclerosis. Sci. Rep..

[264] Hartley M.D., Banerji T., Tagge I.J., Kirkemo L.L., Chaudhary P., Calkins E., Galipeau D., Shokat M.D., DeBell M.J., Van Leuven S. (2019). Myelin repair stimulated by CNS-selective thyroid hormone action. JCI Insight.

[265] Calza L., Fernandez M., Giuliani A., Aloe L., Giardino L. (2002). Thyroid hormone activates oligodendrocyte precursors and increases a myelin-forming protein and NGF content in the spinal cord during experimental allergic encephalomyelitis. Proc. Natl. Acad. Sci. USA.

[266] Baldassarro V.A., Quadalti C., Runfola M., Manera C., Rapposelli S., Calzà L. (2023). Synthetic Thyroid Hormone Receptor-β Agonists Promote Oligodendrocyte Precursor Cell Differentiation in the Presence of Inflammatory Challenges. Pharmaceuticals.

[267] Chaudhary P., Marracci G.H., Calkins E., Pocius E., Bensen A.L., Scanlan T.S., Emery B., Bourdette D.N. (2021). Thyroid hormone and thyromimetics inhibit myelin and axonal degeneration and oligodendrocyte loss in EAE. J. Neuroimmunol..

[268] Rajabinejad M., Asadi G., Ranjbar S., Afshar Hezarkhani L., Salari F., Gorgin Karaji A., Rezaiemanesh A. (2020). Semaphorin 4A, 4C, and 4D: Function comparison in the autoimmunity, allergy, and cancer. Gene.

[269] Smith E.S., Jonason A., Reilly C., Veeraraghavan J., Fisher T., Doherty M., Klimatcheva E., Mallow C., Cornelius C., Leonard J.E. (2015). SEMA4D compromises blood-brain barrier, activates microglia, and inhibits remyelination in neurodegenerative disease. Neurobiol. Dis..

[270] Way S.W., Podojil J.R., Clayton B.L., Zaremba A., Collins T.L., Kunjamma R.B., Robinson A.P., Brugarolas P., Miller R.H., Miller S.D. (2015). Pharmaceutical integrated stress response enhancement protects oligodendrocytes and provides a potential multiple sclerosis therapeutic. Nat. Commun..

[271] Yang Y., Suo N., Cui S.H., Wu X., Ren X.Y., Liu Y., Guo R., Xie X. (2024). Trametinib, an anti-tumor drug, promotes oligodendrocytes generation and myelin formation. Acta Pharmacol. Sin..

